# Eradication of solid tumors by chemodynamic theranostics with H_2_O_2_-catalyzed hydroxyl radical burst

**DOI:** 10.7150/thno.49277

**Published:** 2021-01-01

**Authors:** Nana Wang, Qin Zeng, Ruijing Zhang, Da Xing, Tao Zhang

**Affiliations:** 1 MOE Key Laboratory of Laser Life Science & Institute of Laser Life Science, College of Biophotonics, South China Normal University, Guangzhou, 510631, P.R. China; 2 Guangdong Provincial Key Laboratory of Laser Life Science, College of Biophotonics, South China Normal University, Guangzhou, 510631, P.R. China

**Keywords:** chemodynamic theranostics, activatable, reactive oxygen species, Fenton reaction, multimodal imaging

## Abstract

Activatable theranostics, integrating high diagnostic accuracy and significant therapeutic effect, holds great potential for personalized cancer treatments; however, their chemodynamic modality is rarely exploited. Herein, we report a new *in situ* activatable chemodynamic theranostics PAsc/Fe@Cy7QB to specifically recognize and eradicate cancer cells with H_2_O_2_-catalyzed hydroxyl radical (•OH) burst cascade.

**Methods:** The nanomicelles PAsc/Fe@Cy7QB were constructed by self-assembly of acid-responsive copolymers incorporating ascorbates and acid-sensitive Schiff base-Fe^2+^ complexes as well as H_2_O_2_-responsive adjuvant Cy7QB.

**Results:** Upon systematic delivery of PAsc/Fe@Cy7QB into cancer cells, the acidic microenvironment triggered disassembly of the nanomicelles. The released Fe^2+^ catalyzed the oxidation of ascorbate monoanion (AscH^-^) to efficiently produce H_2_O_2_. The released H_2_O_2_, together with the endogenous H_2_O_2_, could be converted into highly active •OH *via* the Fenton reaction, resulting in enhanced Fe-mediated T_1_ magnetic resonance imaging (MRI). The synchronously released Cy7QB was activated by H_2_O_2_ to produce a glutathione (GSH)-scavenger quinone methide to boost the •OH yield and recover the Cy7 dye for fluorescence and photoacoustic imaging.

**Conclusion:** The biodegradable PAsc/Fe@Cy7QB designed for tumor-selective multimodal imaging and high therapeutic effect provides an exemplary paradigm for precise chemodynamic theranostic.

## Introduction

Nanotheranostics, the integration of multimodal diagnostic and therapeutic modalities into a single system, provides opportunities for nanomedicine-based personalized precision treatments 1-6. In contrast to the conventional nanotheranostics with “always-on” diagnostic signals and pharmacological effects 7-11, the activatable, in particular the stimuli-responsive, materials have attracted great focus for precision medicine 12-19. These nanomaterials can sense endogenous 20 or exogenous stimuli 21, such as pH changes 22, different enzyme levels 23-25, reactive oxygen species (ROS) 26, laser irradiation 27, 28, or temperature changes 29 and are relatively safe and usually non-toxic. Therefore, enhancement of the diagnostic accuracy towards tumor microenvironments as well as the latent therapeutic effects are essential requirements of these nanomaterials designed for the improved treatment efficacy 30, 31.

Among the tumor-related stimuli, ROS species have been viewed as targets for cancer diagnosis and therapy because of high ROS levels in cancer cells 32-36. Recently, chemodynamic therapy (CDT) that can generate highly cytotoxic hydroxyl radicals (•OH) from hydrogen peroxide (H_2_O_2_) *via* the Fenton reaction (Fenton-like) by catalyzing ferrous ion (Fe^2+^) or other Fenton-like ions, has been demonstrated to have potential for cancer treatment 37-44. However, CDT's limitations lie in the poor tumor selectivity and low intratumoral •OH yields 45, 46. Furthermore, the efficacy of CDT is reduced because of the relatively low cellular concentrations of H_2_O_2_ and Fe^2+^, decreasing Fenton reaction efficiency and •OH yield 47-49 as well as clearance of •OH by glutathione (GSH)-abundant antioxidation systems 50-53. Therefore, supplementation of additional CDT agents or decreasing GSH concentration should be effective strategies for enhancing the Fenton reaction efficiency and accelerating toxic •OH generation. Despite significant improvement observed using inorganic nanotheranostic systems 54, 55, such as MnO_2_/GOX and Fe_3_O_4_/GOx 35, 38, 47, 49, the single CDT was still not effective against the localized solid tumors 56-58. In this context, engineered biodegradable chemodynamic theranostics with tumor-selective multimodal imaging and significant in-situ yield of •OH could indisputably enhance the treatment accuracy and efficacy.

In view of the above concerns, herein, we developed theranostic nanomicelles (PAsc/Fe@Cy7QB) with tumor-selective imaging, affording precise and effective eradication of the localized solid tumor *via* an H_2_O_2_-catalyzed •OH burst cascade. Briefly, the polymeric matrix (PAsc-PSFe) was synthesized by copolymerization of ascorbate (polymerized ascorbic acid, PAsc) 59, pH-sensitive segments of Schiff base embedded with Fe^2+^ (polymerized Schiff base-Fe^2+^ complex, PSFe), and 2-(diisorpropylamino) ethyl methacrylate (polymerized diisorpropylamino, PDPA). This complex could be packed into a hydrophobic core due to its intrinsic hydrophobicity, and become hydrophilic after decomposition of the Schiff base and protonation of amine groups in an acidic environment 60, 61. The PAsc/Fe@Cy7QB nanomicelles were obtained after encapsulating a newly synthesized H_2_O_2_-responsive probe (Cy7QB, a Cy7 dye incorporated with the quinone methide-generating boronate ester) within the hydrophobic core of PAsc-PSFe.

**Scheme 1:**
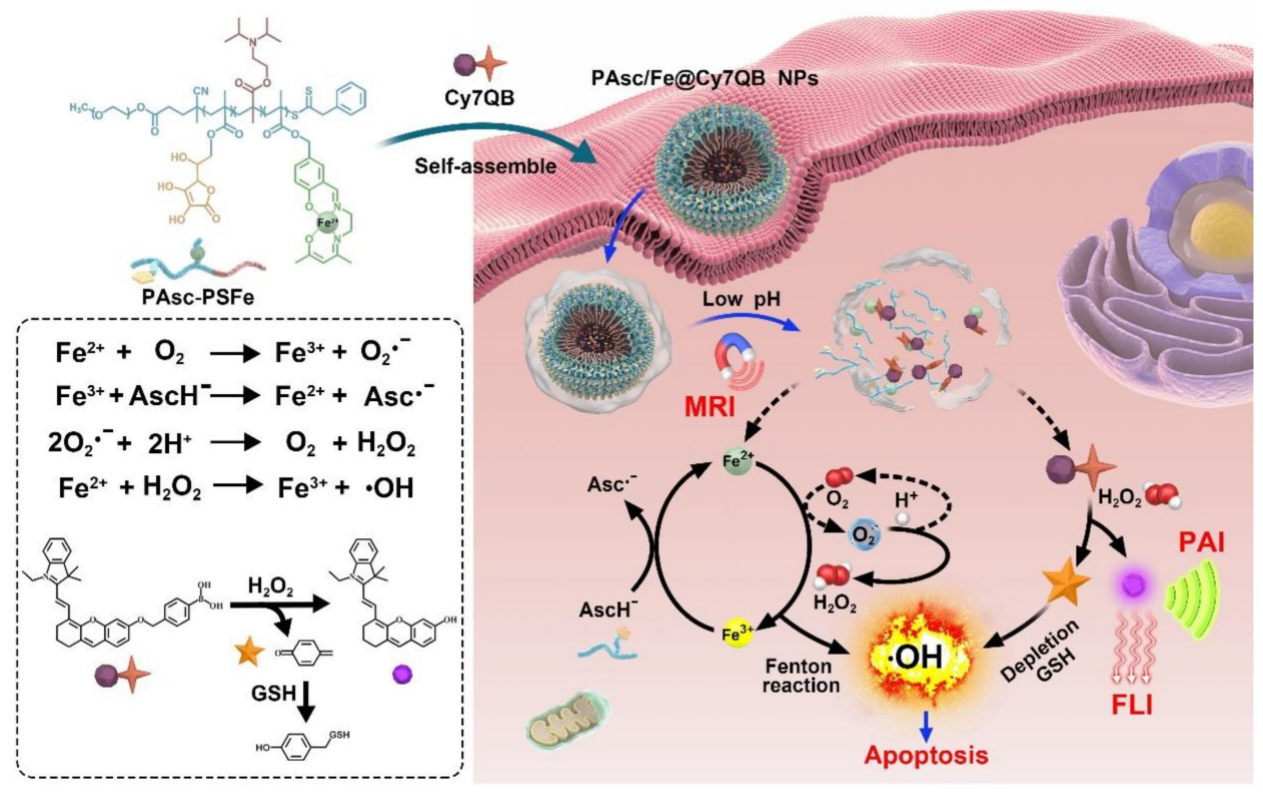
Fabrication procedure and scheme of the theranostic mechanism of PAsc/Fe@Cy7QB for MRI/FLI/PAI multi-modal imaging and the H_2_O_2_-catalyzed •OH burst cascade.

As illustrated in Scheme 1, after systematic administration, the PAsc/Fe@Cy7QB nanomicelles accumulated at the tumor site through the enhanced permeability and retention (EPR) effect, then internalized and endocytosed by tumor cells. The acidic environment of endosomes/lysosomes could subsequently induce decomposition of the Schiff base and PDPA protonation, leading to free Fe^2+^, ascorbate monoanion (AscH^-^) and the adjuvant Cy7QB to the cytoplasm. At this stage, the Fe-mediated T_1_-magnetic resonance imaging (MRI) signal was enhanced dramatically along with the release of Fe^2+^. Notably, the Fe^2+^ could catalyze the oxidation of AscH^-^ into ascorbate radical (Asc^•**-**^) to produce H_2_O_2_, which, along with the endogenous H_2_O_2_, could be efficiently converted into highly active •OH *via* the Fenton reaction. Further, the adjuvant Cy7QB could also be activated by H_2_O_2_ to generate the GSH-scavenger quinone methide and enhance •OH yield. The concomitantly generated dye Cy7 could be used as an imaging agent for fluorescence imaging (FLI) and photoacoustic imaging (PAI) to monitor the process. Thus, stimuli-activatable chemodynamic therapeutics are promising alternatives for tumor therapy, as they can significantly improve the bioavailability of therapeutic agents and inhibit tumor growth with high accuracy and minor side effects *in vivo*.

## Materials and Methods

### Materials

All chemicals were of reagent grade and used without further purification. Poly(ethylene glycol) methyl ether (mPEG_113_-OH) and 2-(diisorpropylamino) ethyl methacrylate were purchased from Sigma-Aldrich Corporation (MO, USA). Diethyl ether, ethyl acetate and all other reagents were acquired from Sinopharm Chemical Reagent Co. Ltd (Shanghai, China). Fetal bovine serum (FBS), penicillin, streptomycin and Dulbecco's modified Eagle's medium (DMEM) were obtained from Thermo Fisher Scientific Co. Ltd (Beijing, China). Catalase (CAT) and phorbol 12-myristate 13-acetate (PMA) were purchased from Aladdin Reagent, Ltd (Shanghai, China). 2,7-dichlorofluorescein diacetate (DCFH-DA) was obtained from Beyotime Biotechnology (Shanghai, China). 3-(4,5-dimethylthiazol-2-yl)-2,5-diphenyltetrazolium bromide (MTT) was acquired from Dojindo Laboratories (Kumamoto, Japan), 4-nitrophthalonitrile fluorescein isothiocyanate (FITC), calcein-acetylmethoxylate (calcein-AM), and propidium iodide (PI) were obtained from Sigma-Aldrich Corporation (MO, USA). All aqueous solutions were prepared in ultrapure water, obtained from a Millipore MilliQ water purification system (Millipore, Billerica, MA USA).

### Characterizations

The optical characteristics of PAsc/Fe@Cy7QB and Cy7QB were investigated using UV/visible absorption spectroscopy (Lambda-35 UV/visible spectrophotometer, Perkin-Elmer, Waltham, MA, USA). Fluorescence spectra of PAsc/Fe@Cy7QB and Cy7QB were acquired on an LS-55 fluorescence spectrophotometer (Perkin-Elmer). Transmission electron microscopy (TEM) images were collected under a field emission high-resolution 2100 F transmission electron microscope (JEOL, Japan) operating at an acceleration voltage of 200 kV. The size of the nanomicelles was measured using a ZEN3690 zetasizer (Malvern, USA).

### Synthesis of compound PAsc-PSFe

FeCl_2_ (20.28 mg, 0.16 mmol) in 50 mL of ethanol was added dropwise to a solution of 150 mg PAsc-PS in 50 mL of ethanol, and stirred in a round-bottomed flask. To avoid oxidation of Fe^2+^, a few drops of glacial acetic acid were added. The resulting solution was magnetically stirred for 12 h under nitrogen at 40 °C, and then the solution was evaporated at 25 °C. The precipitated complex was filtered off, washed with ether, recrystallized from ice-cold ethanol, and dried in air. The optimized copolymer PDPA_36_-b-(PAsc_0.82_-PSFe_0.18_)_65_ (named PAsc-PSFe) was analyzed by ^1^H NMR in d^6^-DMSO. The copolymer PAsc_53_-PDPA_36_ (named PAsc-PDPA) with copolymerization of ascorbate and 2-(diisorpropylamino) ethyl methacrylate was synthesized according to a similar procedure and analyzed by ^1^H NMR in d^6^-DMSO. The copolymer PSFe_12_-PDPA_36_ (named PSFe-PDPA) with copolymerization of Schiff base-Fe^2+^ and 2-(diisorpropylamino) ethyl methacrylate was synthesized using a similar procedure and determined by ^1^H NMR in d^6^-DMSO.

### Preparation of PAsc/Fe@Cy7QB

The Cy7QB was synthesized according to a similar protocol previously described and successfully characterized by ^1^H NMR 62. Subsequently, Cy7QB (2 mg) dissolved in CH_2_Cl_2_ was added to deionized water (2 mL) solution of PAsc-PSFe (10 mg). The mixture was stirred at 25 °C for 4 h. PAsc/Fe@Cy7QB was obtained after dialysis in a cellulose dialysis bag (MWCO 3500 Da) overnight. The resulting solution was freeze-dried for 6 h, and the dry product was re-dispersed in deionized distilled water (pH 7.4) to obtain the PAsc/Fe@Cy7QB solution, which was passed through a 0.22 μm filter to sterilize the sample before use in cultured cells and mice.

### H_2_O_2_ production in solution

The H_2_O_2_ concentration was measured by UV-vis absorbance of oxidized 3,3′,5,5′-tetramethylbenzidine (TMB) in the presence of horseradish peroxidase (HRP). Briefly, HRP (150 mU/mL), and TMB (100 μM) were added to Fe^2+^ (0.16 mM)/ascorbate (0, 0.2, 0.4, 0.6, 0.8 mM), or only ascorbate (0.1, 0.2, 0.4, 0.6, 0.8 mM) solutions at pH 7.4 or 5.0, followed by incubation at 37 °C in the dark. Time-dependent UV-vis spectra were recorded. The characteristic absorbance of oxidized TMB at 370 nm was used to quantify the concentration of H_2_O_2_ based on the standard curve. Copolymers PAsc-PSFe, PAsc-PDPA and PSFe-PDPA were also treated similarly.

### •OH production in solution

The amount of •OH was determined by the •OH-specific fluorescence turn-on probe benzoic acid (BA). Briefly, BA (10 µM), Fe^2+^ (0.16 mM) and ascorbate (0, 0.2, 0.4, 0.6, 0.8 mM) were added to PBS (pH 7.4 or 5.0) at 37 °C. Subsequently, the fluorescence spectra of the solution were recorded at determined time intervals and the emission intensity at 421 nm was plotted as a function of incubation time. PAsc/Fe@Cy7QB, PAsc-PSFe, PAsc-PDPA and PSFe-PDPA were also treated similarly.

### Cell culture and confocal fluorescence imaging

Human hepatoma cells (HepG2) were cultured in DMEM containing 10% FBS (γ-irradiated and sterile-filtered) and 1% penicillin/streptomycin at 37 °C in a humidified atmosphere containing 5% of CO_2_. HepG2 tumor cells were seeded in 35 mm confocal culture dishes and allowed to grow for 24 h. Cells were incubated for 2 h, the culture media were removed, and the cells were washed with PBS. Confocal fluorescence imaging analyses were performed using a confocal laser scanning microscope (ZEISS LSM 510 META, Germany). PAsc/Fe@Cy7QB was excited at 680 nm and emission was recorded at 690-730 nm.

### Cell uptake

Cell phagocytosis was assessed by confocal laser scanning microscopy (CLSM). HepG2 cells were incubated with PAsc/Fe@Cy7QB (50 μg/mL) for various durations (0, 0.5, 1 and 2 h). The uninternalized nanomicelles were removed by washing several times with PBS.

### Cellular ROS detection

The culture medium of HepG2 cells was replaced with fresh DMEM, followed by the addition of PAsc/Fe@Cy7QB, PAsc-PSFe, PSFe-PDPA and PAsc-PDPA (50 μg/mL). After 8 h, the medium was removed and the cells were washed with PBS several times. Subsequently, 10 μM of the fluorescent probe 2,7-dichlorofluorescein diacetate (DCFH-DA) was added for 30 min to react with ROS and generate the fluorescent product DCF, which could be observed by CLSM.

### Cellular GSH levels

A group of HepG2 cells were treated with 0, 12.5, 25, 50 μg/mL of PAsc/Fe@Cy7QB for 2 h. Another group was treated with catalase (CAT) before treatment with PAsc/Fe@Cy7QB (50 μg/mL). The cells were harvested, washed, and then lysed on ice in 40 μL of Triton X-100 lysis buffer. After 20 min, lysates were centrifuged and 10 μL of the supernatant was mixed with 50 μL of Ellman's reagent (0.5 mM DTNB). GSH concentration was determined by measuring the absorbance at 405 nm using a microplate reader. The levels of GSH in the treated cells were compared with the basal GSH levels in untreated cells.

### *In vitro* cell viability assay

The cytotoxicity was studied by the 3-(4,5-dimethylthiazol-2-yl)-2,5-diphenyltetrazolium bromide (MTT) assay. HepG2 cells were seeded in a 96-well plate and cultured in 100 µL DMEM medium for 24 h at 37 °C. Subsequently, the medium was replaced with 200 µL of fresh medium containing different concentrations of the PAsc-PDPA, PSFe-PDPA, PAsc-PSFe or PAsc/Fe@Cy7QB. After incubation for another 24 h, MTT (20 µL, 5 mg/mL) was added and incubated for 4 h to produce purple formazan. The supernatant was then replaced with 150 µL of DMSO. The optical density (OD) was monitored using a microplate reader at 570 nm. Cell viability (%) was determined by comparing the absorbance (570 nm) of the treated cells with that of untreated cells. Five independent experiments were performed for each test.

### Tumor mouse model

All animal procedures were performed in accordance with the National Institutes of Health (NIH) Guidelines for the Care and Use of Laboratory Animals of South China Normal University, and the experiments were approved by the Animal Ethics Committee of South China Normal University. 4-week-old female BALB/c nude mice were purchased from the Animal Experiment Center, Southern Medical University, and bred in an axenic environment. HepG2 tumor cells (1.0 × 10^6^ cells) in PBS (100 μL) were subcutaneously injected into the flanks of each mouse. The animals were subjected to the treatments when the tumors reached approximately 100 mm^3^. The tumor volumes were calculated using the following formula: tumor volume = the greatest longitudinal diameter (length) × the greatest transverse diameter (width)^2^ × 0.5.

### *In vivo* pH-responsive MRI

For *in vivo* T_1_-weighed MRI, PAsc/Fe@Cy7QB (100 μL, 50 μg/mL) was intravenously injected into HepG2 tumor-bearing BALB/c nude mice. After 0, 2, 4, 8, 12, and 24 h, the MRI of tumor tissues was recorded by MRI BioSpin ICON 1T (Bruker) with a small animal specific body coil. The relative MRI signal intensity was measured using Image J software.

### *In vivo* FLI and PAI imaging

PAsc/Fe@Cy7QB (100 μL, 50 μg/mL) were injected into the tail veins of the HepG2 tumor-bearing mice, which were imaged and analyzed at 0, 2, 4, 8, 12, and 24 h after injection with an NIR imaging system (Odyssey LI-COR, USA). The fluorescence signals were collected at λ_em_ = 710 nm under excitation with a 680 nm continuous laser. For PAI, the mice were pretreated with saline, catalase (CAT), or phorbol 12-myristate 13-acetate (PMA) for 24 h. PAI was performed by a PA computed tomography system equipped with a 10 MHz, 10 mJ/cm^2^, 384 element ring ultrasound array, and an optical parametric oscillator (OPO) (Surelite II-20, Continuum, Santa Clara, CA, USA) with 4-6 ns pulse duration and 20 Hz pulse repetition rate light source.

### Blood circulation

The HepG2 tumor-bearing mice were intravenously injected with PAsc/Fe@Cy7QB (100 μL, 50 μg/mL), and approximately 20 μL blood was collected at 0, 2, 4, 8, 12, 24, and 48 h post-injection to which 980 μL of physiological saline containing 10 mM EDTA anticoagulant. Blood concentration of PAsc/Fe@Cy7QB was then determined by measuring the characteristic fluorescence of Cy7. The curve of blood terminal half-life of PAsc/Fe@Cy7QB was fitted based on the single-compartment pharmacokinetic model.

### *In vivo* biodistribution

HepG2 tumor-bearing mice were intravenously injected with PAsc/Fe@Cy7QB (100 μL, 50 μg/mL) for biodistribution measurement, and then the mice were sacrificed at various time points post-injection (each group contained three mice). The major organs of mice were collected at 8, 12, 24, 48, and 72 h after the injection and were solubilized by tissue lysate for fluorescence measurement to determine Cy7 content.

### *In vivo* excretion study

Mice were intravenously injected with PAsc/Fe@Cy7QB (100 μL, 50 μg/mL). Urine and feces of each mouse were then collected at various time points (12, 24, 48, 72, 96, and 120 h) and solubilized in 10% DMSO in PBS. The absorbance at 710 nm was measured to determine the percentage of injected dose per gram of urine and feces.

### Hemolysis tests

Whole blood was collected from mice in heparinized-tubes and centrifuged at 3000 rpm for 5 min. The pellet was washed three times with PBS by centrifugation and resuspended in the same buffer. This suspension of red blood cells was always freshly prepared and used within 48 h after collection. Subsequently, 500 mL of 2% erythrocytes (*v*/*v*) were mixed with 500 mL of 20, 40, 60, 80, or 100 μg/mL PAsc/Fe@Cy7QB solution and incubated at 37 °C for various times 63-65. Erythrocytes mixed with pure water were used as 100% hemolysis. The samples were centrifuged and the absorbance of the supernatants at 540 nm was measured. The percentage of hemolysis was calculated using the formula 66: Hemolysis (%) = (I/I_0_) × 100%, where I represents the absorbance of the supernatant for erythrocyte suspension with PAsc/Fe@Cy7QB, and I_0_ represents the absorbance of completely hemolyzed erythrocytes in pure water.

### Antitumor activity

For the evaluation of the antitumor activity against HepG2 tumors, PBS, PAsc-PDPA (200 μL, 50 μg/mL), PSFe-PDPA (200 μL, 50 μg/mL), PAsc-PSFe (200 μL, 50 μg/mL), and PAsc/Fe@Cy7QB (200 μL, 50 μg/mL) were intravenously injected after the tumor volume reached ~100 mm^3^. Tumor volumes were monitored every two days using a caliper to measure the perpendicular diameter of the tumors. The individual tumor volume (V) was calculated using the following equation: V = X × Y^2^/2, where X and Y represent the longest and shortest diameters of the tumors. Typical images of HepG2 tumors were obtained using a digital camera. The body weight of each mouse was recorded every two days.

### Histologic examination

Hematoxylin and eosin (H&E) staining was performed according to the method provided by the vendor (BBC Biochemical). After the experiments were completed, the mice were sacrificed. The major organs (liver, lungs, kidneys, heart, and spleen) and tumor tissues from the mice of the five groups were retrieved, cut into 4-μm-thick sections, fixed in a 10% paraformaldehyde solution for 8 h at room temperature, followed by dehydration with ethanol, and then processed routinely into paraffin. The sliced tissues were stained with H&E and examined using an inverted fluorescence microscope system (Nikon E 200).

### Statistical analysis

Data analyses were performed using the Prism GraphPad software (Version 7.0, GraphPad Software, San Diego, CA). Statistical analyses were performed according to a t-test. In all cases, * was used for p *<* 0.05, **for p *<* 0.01, and *** for p *<* 0.001. Values were expressed as the mean ± SD. All experiments were performed at least in triplicates. The samples/animals were randomly allocated to the experimental group.

## Results and Discussion

### Molecular design principle and preparation of PAsc/Fe@Cy7QB nanomicelles

Ascorbate is recognized as an antioxidant or a pro-oxidant 67. Generally, the endogenous ascorbate is thought to be an excellent reducing agent by donating one or two electrons to protect biomolecules from oxidative damage 68. However, ascorbate could also widely exert pro-oxidant effects in the presence of transition metal ions 69-71. The combination of ascorbate and iron has been used as an oxidizing system for the hydroxylation of alkanes, aromatics, and other oxidants 70, 72. The mechanism 73, 74 is summarized in Scheme 1 and Figure S1: the oxidation of ascorbate is catalyzed by Fe^2+^ to produce the Asc^·-^ and superoxide radical (O_2_^·-^), Reaction (1) + (2); the O_2_^·-^ in turn results in H_2_O_2_ and O_2_ dismutation, Reaction (3); Fe^2+^ reacts with H_2_O_2_ to generate Fe^3+^ and •OH in the classic Fenton Reaction (4).

Herein, we designed an *in situ* activatable theranostic system for H_2_O_2_-catalyzed •OH burst cascade to selectively enhance CDT's efficacy against cancers. The all-in-one nanomicelles PAsc/Fe@Cy7QB were constructed from acid-responsive polymersomes incorporating ascorbate and Schiff base-Fe^2+^ complex and Cy7QB in the membranes and inner aqueous cavities, respectively. First, the optimal ratio of ascorbate and Fe^2+^ was determined using BA to monitor the production of •OH *via* the Fenton reaction (Figure S2) 74. Subsequently, the optimized copolymer PAsc-PSFe was synthesized *via* copolymerization of the ascorbate and Schiff base-Fe^2+^ complex from the mPEG_113_-OH as a micelle matrix for the self-produced H_2_O_2_ and acid-triggered Fe^2+^ release (Figure S3), and the degrees of polymerization were determined by ^1^H NMR (Figure S4). For comparison, copolymer PAsc-PDPA polymerized with ascorbate and copolymer PSFe-PDPA polymerized with Schiff base-Fe^2+^ complex were also synthesized and characterized by ^1^H NMR (Figure S5-6). To avoid the clearance of •OH by GSH, the H_2_O_2_-activatable probe (Cy7QB), a GSH-scavenging adjuvant was synthesized and characterized by incorporating the quinone methide-generating moiety boronate ester into Cy7 75 (Figure S7-8). The final nanomicelles were then successfully obtained by self-assembly of PAsc-PSFe and Cy7QB; the loading capacity was measured as 84.15% (Figure S9) and the critical micelle concentration (CMC) was 30.12 µg/mL 76, 77 (Figure S10).

### Characterization of PAsc/Fe@Cy7QB

**Figure 1:**
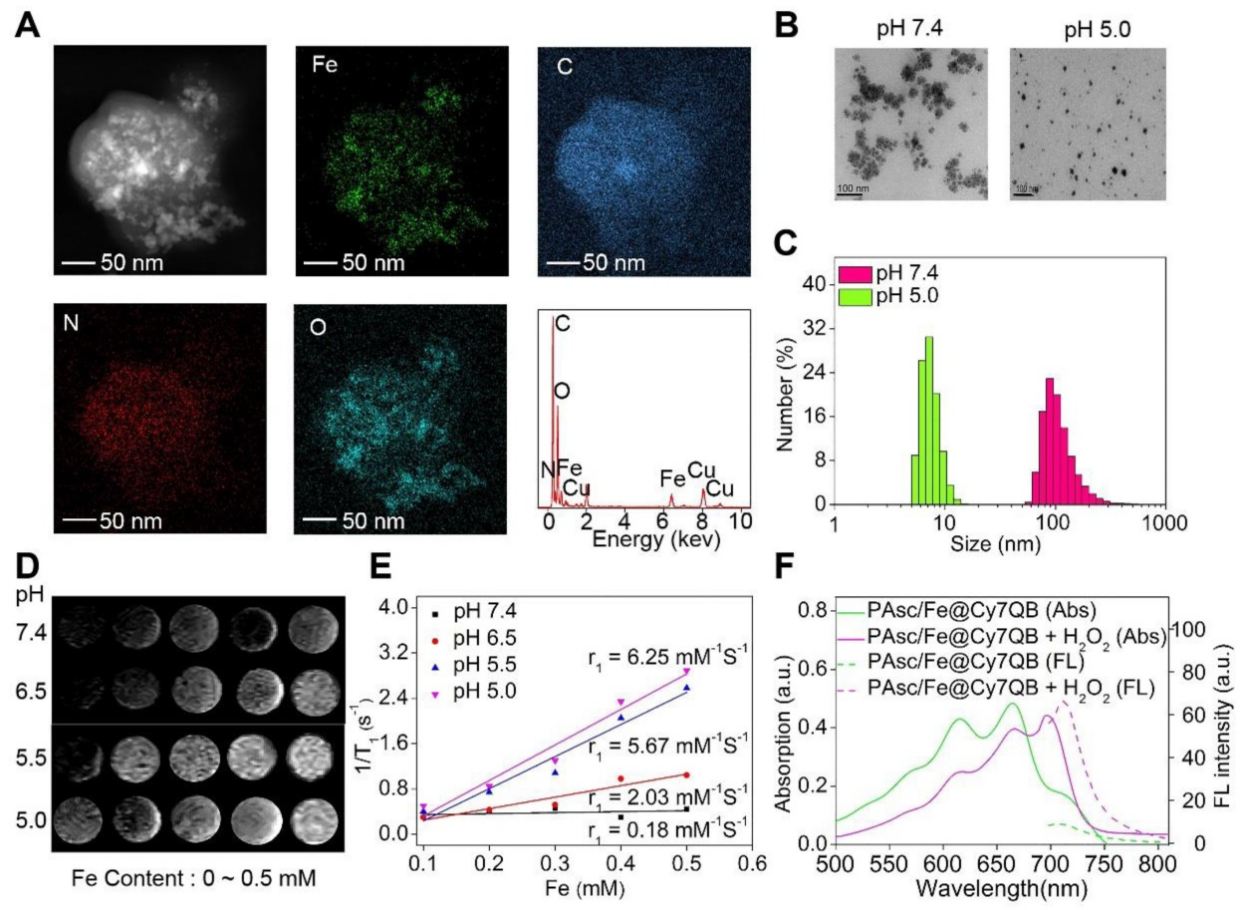
Characterization of PAsc/Fe@Cy7QB. (A) EDS elemental mappings and corresponding spectra of PAsc/Fe@Cy7QB. (B) TEM images and (C) Size distribution of PAsc/Fe@Cy7QB at pH 7.4 and pH 5.0. (D) T_1_-weighted MRI of the PAsc/Fe@Cy7QB was recorded using MR scanner at different Fe concentrations (0 ~ 0.5 mM) and pH values (7.4, 6.5, 5.5, and 5.0). (E) Corresponding r_1_ value. (F) Absorption and fluorescence spectra of PAsc/Fe@Cy7QB (50 μg/mL, pH 5.0) in PBS at indicated varied concentrations of H_2_O_2_ (0 ~ 10 eq).

The energy dispersive spectrometer (EDS)-elemental mappings and spectrum revealed that PAsc/Fe@Cy7QB consisted of Fe, C, O, and N elements (Figure 1A), confirming the chemical composition of PAsc/Fe@Cy7QB. TEM and dynamic light scattering (DLS) revealed that PAsc/Fe@Cy7QB had well-dispersed spherical morphology under physiological pH (pH 7.4) (Figure 1B-C). Under these conditions, the hydrodynamic size of the nanomicelles was almost unchanged in 48 h, indicating good stability (Figure S11). As a characteristic feature of solid tumors, high glycolytic activity facilitates lactate and H^+^ secretion, making the tumor microenvironment acidic 78. Under acidic conditions, the average hydrodynamic diameter of PAsc/Fe@Cy7QB was shortened from 110 ± 2 nm at pH 7.4 to 8 ± 2 at pH 5.0, apparent changes in their morphology were also observed due to the degradation of the acid-sensitive polymer (Figure 1B-C, S12-13). More importantly, T_1_-weighted MRI became gradually brighter from pH 7.4 to pH 5.0, and the longitudinal relativities r_1_ at pH 5.0 were 30-fold higher than at pH 7.4 because of the Fe^2+^ release from Schiff base under acidic conditions (Figure 1D-E). The release of Fe^2+^ from the PAsc-PSFe polymer under acidic conditions was confirmed using the standard potassium ferricyanide and potassium thiocyanate method (Figure S14).

The synchronous release of Cy7QB was also confirmed by UV-vis absorption and emission spectra, consistent with the sensing performance with the free Cy7QB (Figure 1F). Due to the significant absorption responsiveness of PAsc/Fe@Cy7QB towards H_2_O_2_, the fluorescence intensity gradually increased after the addition of H_2_O_2_ (Figure S15). The PA signal of PAsc/Fe@Cy7QB also showed a positive linear correlation with H_2_O_2_ concentration (Figure S16). Altogether, our results indicated that the nanomicelles PAsc/Fe@Cy7QB could disassemble in an acidic microenvironment to release Fe^2+^, AscH^-^, and the molecular adjuvant Cy7QB, accompanied by remarkable enhancement of MR signals and recovery of the H_2_O_2_-sensitive fluorescent and photoacoustic signals, facilitating the multimodal imaging of the localized tumors.

### Generation of H_2_O_2_ and •OH in solution

**Figure 2:**
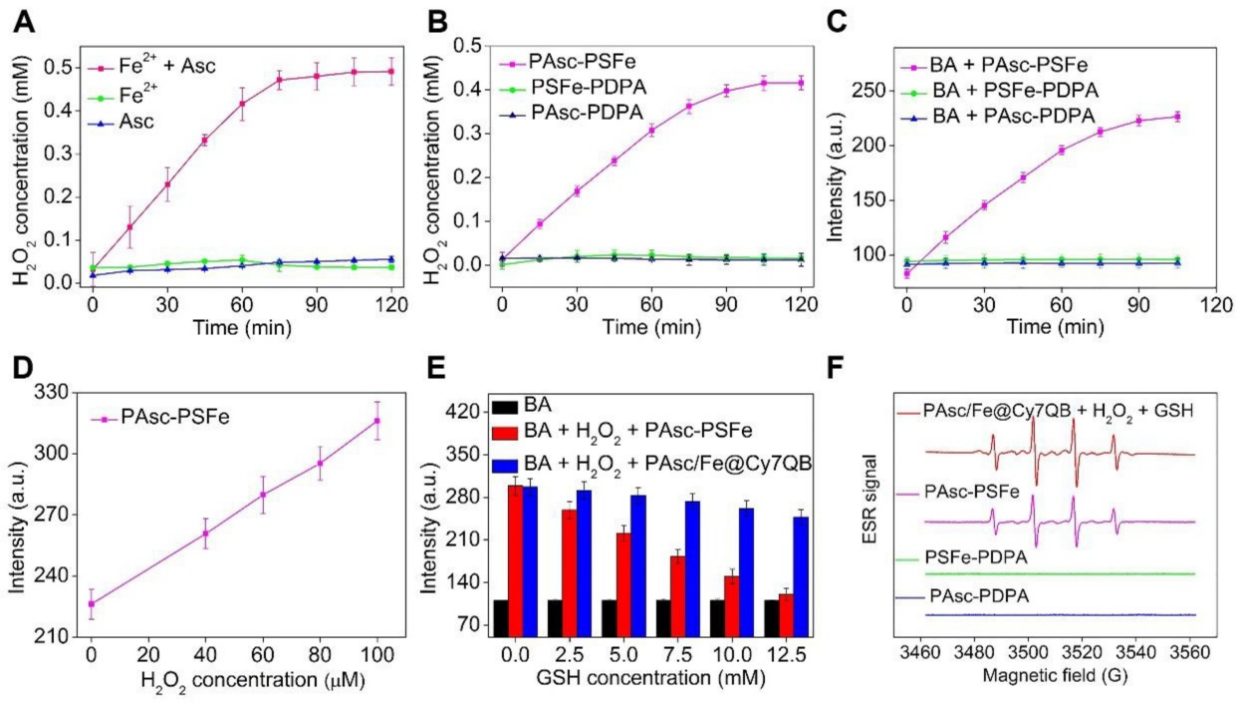
Generation of H_2_O_2_ and •OH in solution. (A-B) Time-dependent H_2_O_2_ production by Fe^2^-mediated Fenton reaction in different solutions. Ascorbate (0.8 mM), Fe^2+^ (0.16 mM). (C) Time-dependent increase in fluorescence using BA as the fluorescence probe for the detection of •OH production. The emission of fluorescence by oxidized BA at 426 nm was used to determine the levels of •OH at pH 5.0. (D) Detection of •OH in samples treated at indicated H_2_O_2_ concentrations with polymer PAsc-PSFe. (E) Fluorescence spectra of BA by •OH generated by different concentrations of GSH-treated with PAsc-PSFe + H_2_O_2_ and PAsc/Fe@Cy7QB + H_2_O_2_ at pH 5.0. (F) ESR spectra of PAsc/Fe@Cy7QB + 100 μM H_2_O_2_ + 10 mM GSH, PAsc-PSFe, PAsc-PDPA, and PSFe-PDPA.

After validation of the acid-triggered degradation of PAsc/Fe@Cy7QB, *in situ* production of H_2_O_2_ was investigated. We utilized TMB and HRP to quantify the H_2_O_2_ concentration by measuring the absorbance of oxidized TMB (Figure S17). H_2_O_2_ concentration reached up to 0.5 mM in the presence of free Fe^2+^ (0.16 mM) and ascorbate (0.8 mM) at pH 5.0 (Figure 2A), and the concentration further increased with the increase in ascorbate (Figure S18). However, H_2_O_2_ generation was negligible after treatment with ascorbate at both pH 7.4 and 5.0 (Figure S19). These results confirmed that the Fe^2+^ could catalyze the oxidation of ascorbate into Asc^·-^ for *in situ* H_2_O_2_ production under acidic conditions 74, 79.

We then investigated the possibility of using PAsc/Fe@Cy7QB to produce H_2_O_2_, and PAsc-PDPA without Schiff base-Fe^2+^ and PSFe-PDPA without ascorbate were chosen as controls to accurately quantify H_2_O_2_ levels. A similar trend was observed for PAsc-PSFe containing ascorbate and Schiff base-Fe^2+^, suggesting that the ascorbate in PAsc-PSFe could induce H_2_O_2_ generation *in situ* at pH 5.0 (Figure 2B, S20). Subsequently, •OH generation with Fe^2+^ and ascorbate and the depletion effect of GSH on •OH at pH 7.4 or 5.0 were investigated. We found that BA's fluorescence intensity gradually increased with the addition of ascorbate in the presence of Fe^2+^ at pH 5.0 within 2 h (Figure S21), indicating efficient generation of •OH. However, the levels of •OH remained almost unchanged after the same treatment at pH 7.4 (Figure S22). PAsc-PSFe was also found to efficiently produce •OH under acidic conditions (Figure 2C, S23). The addition of exogenous H_2_O_2_ could also boost •OH generation (Figure 2D, S24-25), indicating ability of PAsc-PSFe to produce •OH by utilizing both the produced and endogenous cellular H_2_O_2_. However, further investigations showed that BA's fluorescence intensity in PAsc/Fe@Cy7QB at pH 5.0 was 3-fold stronger than PAsc-PSFe in the presence of 10 mM GSH (Figure S26), ascribed to the removal of GSH by PAsc/Fe@Cy7QB *via* the *in situ* generated quinone methide (Figure S27). Overall, acid-degradable PAsc/Fe@Cy7QB, integrating self-catalytic Fenton reactions and the ability to eliminate GSH, could provide a cascade burst of •OH production in the tumor microenvironment for highly efficient and precise eradication of solid tumors (Figure 2E). Furthermore, electron spin resonance (ESR) was utilized to monitor the •OH signals by employing 5,5-dimethyl-1-pyrroline-N-oxide (DMPO) as the spin-trapping agent. The results indicated that •OH was effectively generated by PAsc/Fe@Cy7QB that integrated self-catalytic Fenton reaction and the ability to eliminate GSH under acidic conditions (Figure 2F).

### *In vitro* H_2_O_2_-selective bioimaging and intracellular ROS levels

**Figure 3:**
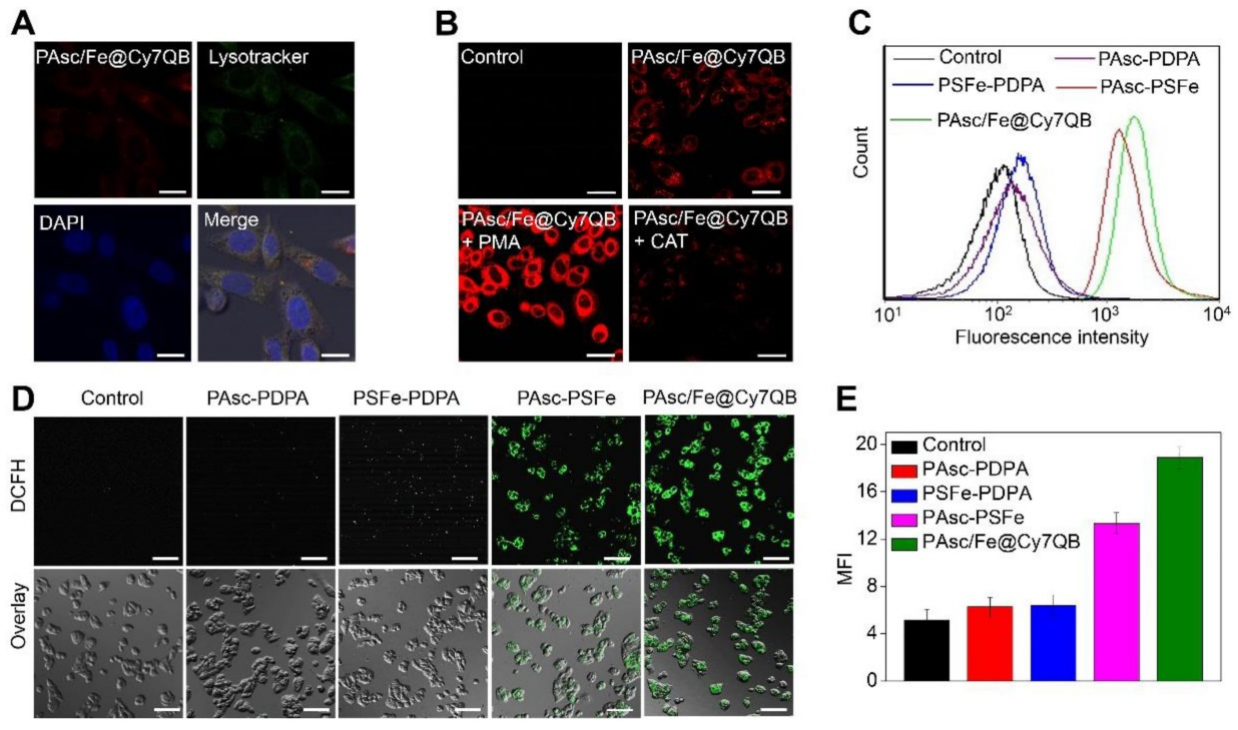
*In vitro* H_2_O_2_-selective bioimaging and intracellular ROS levels. (A) CLSM images showing the intracellular distribution of the PAsc/Fe@Cy7QB. (B) CLSM images of HepG2 cells incubated with 50 μg/mL PAsc/Fe@Cy7QB in the presence or absence of PMA or CAT. (C) Flow cytometry analysis and (D) CLSM images of HepG2 cells after treatment with PAsc/Fe@Cy7QB, PAsc-PSFe, PSFe-PDPA, and PAsc-PDPA for 2 h and subsequent staining with the ROS fluorescence probe DCFH-DA. (E) Corresponding mean fluorescence intensity (MFI) values in (D). All images have a scale bar of 20 μm.

Given the highly effective *in vitro* generation of •OH by PAsc/Fe@Cy7QB under acidic conditions, we explored their responsiveness to H_2_O_2_ and anticancer effects in tumor cells. The fluorescence intensity of HepG2 incubated with PAsc/Fe@Cy7QB increased significantly over time and reached a maximum at 2 h (Figure S28). The appearance of merged yellow dots from green (Lysotracker) and red (PAsc/Fe@Cy7QB) pixels showed that PAsc/Fe@Cy7QB exhibited good colocalization with lysosomes (Figure 3A). The acidic environment of endosomes/lysosomes could induce decomposition and destabilization of the responsive nanomicelles 80, which might explain the disintegration of PAsc/Fe@Cy7QB and release of Fe^2+^, AscH^-^ and the adjuvant Cy7QB in a relatively acidic microenvironment. Subsequently, upregulation of H_2_O_2_ in HepG2 cells following treatment with PMA (1 mg/mL) led to brighter fluorescence imaging. In contrast, H_2_O_2_ depletion following treatment with CAT (10 U/mL) resulted in a decrease in fluorescence intensity, beneficial for monitoring the disintegration and visualizing movement of PAsc/Fe@Cy7QB (Figure 3B, S29).

To understand the intracellular H_2_O_2_-catalyzed toxicity of PAsc/Fe@Cy7QB, ROS levels were further monitored by the probe DCFH-DA 81-83, which can emit green fluorescence after oxidation by ROS. As shown in Figure 3C, flow cytometric analysis verified that •OH generation in the PAsc-PSFe group was higher than the control group, which was ascribed to the self-catalytic Fenton reaction. However, •OH generation in the PAsc/Fe@Cy7QB group was higher than the control group due to the synergistic effect of self-catalytic Fenton reaction and GSH depletion. Similar results were observed by CLSM analysis, further verifying a significant H_2_O_2_-catalyzed •OH burst cascade by PAsc/Fe@Cy7QB treatment (Figure 3D-E). GSH quantification in HepG2 cells treated with PAsc/Fe@Cy7QB confirmed that nanomicelles could decrease the cellular GSH concentration (Figure S30), consistent with the *in vitro* findings. Also, H_2_O_2_ elimination by CAT prevented PAsc/Fe@Cy7QB-induced H_2_O_2_ mediated boronate oxidation to generate quinone methide and recover GSH levels in the cells. Thus, PAsc/Fe@Cy7QB were effectively taken up by tumor cells, producing •OH with high efficiency in the acidic tumor microenvironment to induce significant localized toxicity and exert excellent anticancer effects.

### Assessment of PAsc/Fe@Cy7QB-induced cytotoxicity

**Figure 4:**
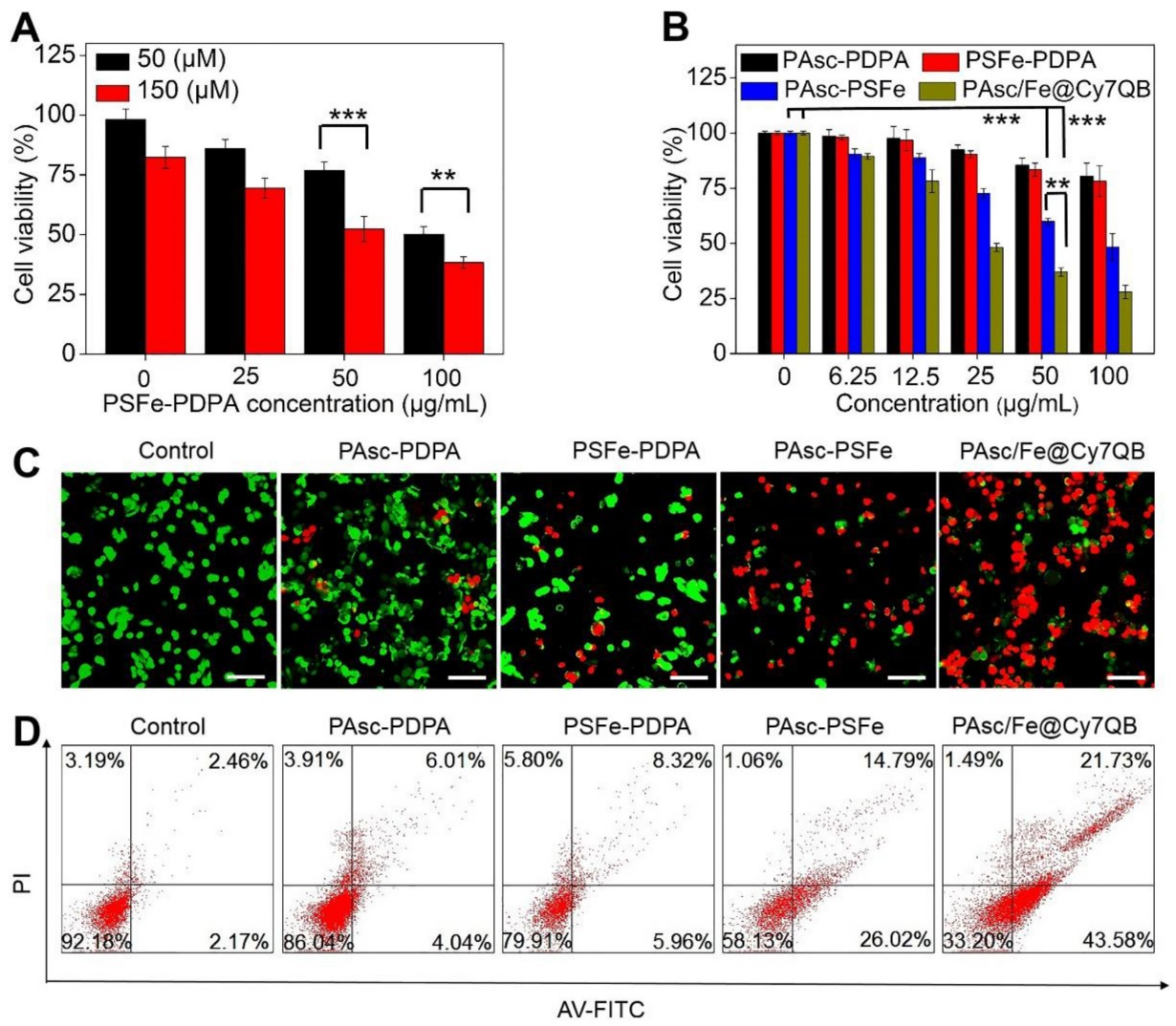
Assessment of PAsc/Fe@Cy7QB-induced cytotoxicity. (A) Cytotoxicity of PSFe-PDPA in the presence of H_2_O_2_ (50 μM or 150 μM). (B) Cytotoxicity against HepG2 cells at various concentrations. (C) The live/dead assay and (D) Annexin V/PI assay was used to evaluate the cytotoxicity after incubation with PBS, PAsc-PDPA, PSFe-PDPA, PAsc-PSFe, or PAsc/Fe@Cy7QB (50 µg/mL). All images have a scale bar of 20 μm. P values were calculated by two-tailed Student's t-test (***p < 0.001, or *p < 0.05).

Encouraged by the high catalytic performance of PAsc/Fe@Cy7QB, we assessed the cell viability by using the standard MTT cell assay. First, treatment of HepG2 cells with PSFe-PDPA at the same concentration of H_2_O_2_ resulted in significant cytotoxicity compared to cells without PSFe-PDPA treatment, demonstrating the stronger cytotoxicity of •OH compared to H_2_O_2_ (Figure 4A). The cytotoxicity of PAsc/Fe@Cy7QB was then explored using PAsc-PSFe, PAsc-P DPA and PSFe-PDPA groups as controls. As shown in Figure 4B, the PAsc/Fe@Cy7QB group exerted significant concentration-dependent cytotoxicity, and the percentage of cell survival following treatment with PAsc/Fe@Cy7QB (26%) was lower than that of PAsc-PDPA (84%) with insufficient intracellular ROS and PAsc-PSFe (49%) with only self-catalytic Fenton reaction. In particular, PSFe-PDPA caused only a slight reduction in cell viability due to the insufficient intracellular H_2_O_2_ levels to produce •OH, which was further confirmed by the negligible cytotoxicity of pure Fe^2+^ (Figure S31). The combination index (CI) value of PAsc-PSFe, and PAsc/Fe@Cy7QB was determined to be 0.51 and 0.47, respectively (Table S1), suggesting that the combined effect led to a stronger inhibition compared with the single composition 84. The high cytotoxicity of PAsc/Fe@Cy7QB was also verified by Calcein AM and propidium iodide (PI) double-staining of liver cells; live cells (green) and dead cells (red) (Figure 4C). Flow cytometric analysis with Annexin V-FITC together with PI as markers provided similar results (Figure 4D). These results confirmed the •OH burst cascade by the self-catalytic Fenton reaction and GSH depletion.

### *In vivo* multimodal imaging

**Figure 5:**
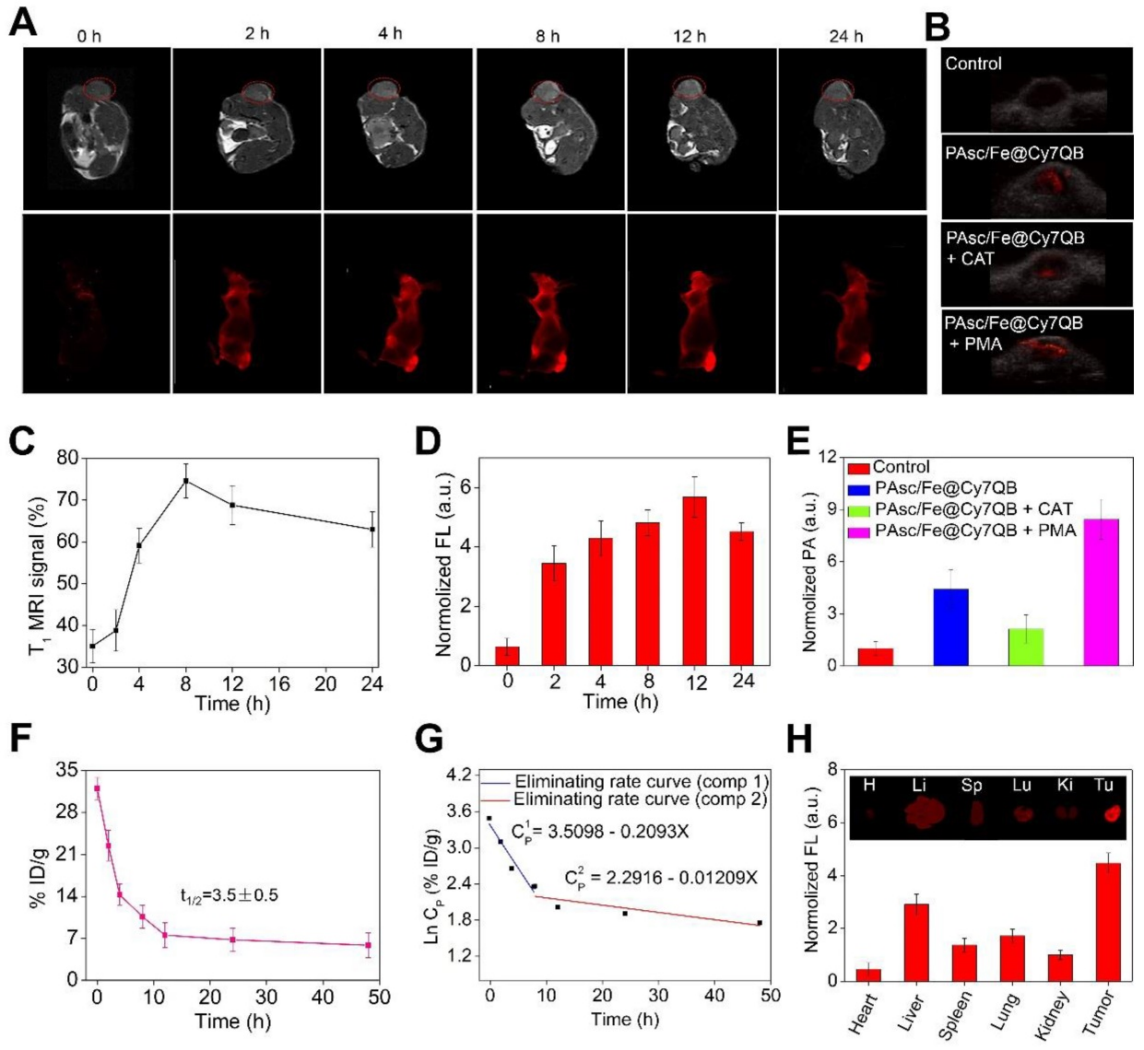
* In vivo* multimodal imaging. (A)* In vivo* MRI/FLI at 0, 2, 4, 8, 12, and 24 h i. v. post-injection of PAsc/Fe@Cy7QB (50 μg/mL, 100 μL). (B) PAI of each group at 12 h after PAsc/Fe@Cy7QB injection *via* the tail vein: PAsc/Fe@Cy7QB only; PAsc/Fe@Cy7QB + CAT; PAsc/Fe@Cy7QB + PMA. (C) Analysis of the tumor region by the MRI signals in (A). (D) Fluorescence intensity in mice after PAA/Fe@Cy7QB injection. (E) Analysis of the tumor region by the PAI signals in (B). (F) Blood level curve of PAsc/Fe@Cy7QB in mice fluorescence measurement of Cy7 in blood at different time points post i.v. injection. (G) Eliminating rate curve of injected PAsc/Fe@Cy7QB in blood circulation according to the Ln (concentration)-time relationship. The two stage eliminating rates of PAsc/Fe@Cy7QB are shown. (H) FLI of major organs and tumors of mice after 24 h i.v. injection PAsc/Fe@Cy7QB.

Multimodal imaging provides the advantages of various imaging methods, such as high-resolution MRI, highly sensitive FLI and high-depth PAI, to facilitate the screening of early micro-tumors and guide precision therapy 85-89. The excellent magnetic, fluorescence, and photoacoustic properties of the PAsc/Fe@Cy7QB *in vitro* prompted us to apply multimodal imaging-guided tumor-targeted *in vivo* CDT. After intravenous (i.v.) injection of PAsc/Fe@Cy7QB (50 μg/mL, 100 μL) into the HepG2 tumor-bearing mouse, MRI of the tumor region was acquired over time and recorded at 0, 2, 4, 8, 12, and 24 h post-injection (Figure 5A). The T_1_-weighted MRI signal was substantially increased from 30% to 75% within 24 h (Figure 5C), confirming efficient tumor accumulation of PAsc/Fe@Cy7QB. Next, NIR fluorescent imaging of HepG2 tumor-bearing mice following PAsc/Fe@Cy7QB injection was performed, as depicted in Figure 5A. The fluorescence in the tumor region was detectable at 2 h post-injection, and its intensity gradually increased reaching a maximum at 12 h post-injection (Figure 5D). After 12 h of i.v injection of PAsc/Fe@Cy7QB, the HepG2 tumor-bearing mice pretreated with saline, CAT, or PMA were monitored by PAI (Figure 5B, E). These results implied that H_2_O_2_ could selectively unlock imaging features of the nanomicelles *in vivo*.

The biodistribution in the main organs and in blood circulation was also measured to determine the *in vivo* behavior of the nanomicelles. The results showed that the blood level of PAsc/Fe@Cy7QB gradually decreased over time but remained at a relatively effective concentration even at 24 h post-injection (Figure 5F). The eliminating rate constants 38, 90 of PAsc/Fe@Cy7QB were calculated to be -0.2093 μg/mL per hour in the first stage and decreased to -0.01209 μg/mL per hour in the second stage with a shifting time interval of 6.18 h (Figure 5G). The tumor as well as all main organs from HepG2 tumor-bearing mice treated with PAsc/Fe@Cy7QB were harvested after 24 h (Figure 5H). FLI indicated that the theranostic PAsc/Fe@Cy7QB specifically accumulated in the tumor region *via* the multi-targeting effect of EPR and subsequent pH-triggered decomposition and H_2_O_2_-driven deboronation reaction. The rapid decrease in PAsc/Fe@Cy7QB levels within 72 h in various organs after post-injection further revealed its desirable good biodegradation (Figure S32).

Although the relatively high PAsc/Fe@Cy7QB accumulation was found in the liver due to the reticuloendothelial system (RES) 91, the tumor selectivity of the nanomicelles was significantly dominant and could be attributed to the multi-targeting effect. The feces and urine were also collected to study the possible clearance pathway of PAsc/Fe@Cy7QB (Figure S33). High levels of Cy7 were detected in both urine and feces, indicating good metabolic characteristics of PAsc/Fe@Cy7QB that could be excreted through both the kidneys and gastrointestinal system. Very low levels of Cy7-species remained in the mouse body after 120 h. The results were consistent with the relatively high accumulation levels of PAsc/Fe@Cy7QB in the liver, spleen and kidney. Taken together, these results indicated that PAsc/Fe@Cy7QB provided outstanding MR/FL/PA multimodal imaging for defining the tumor region and precise guidance for subsequent CDT.

### *In vivo* tumor therapeutic effects of PAsc/Fe@Cy7QB

**Figure 6:**
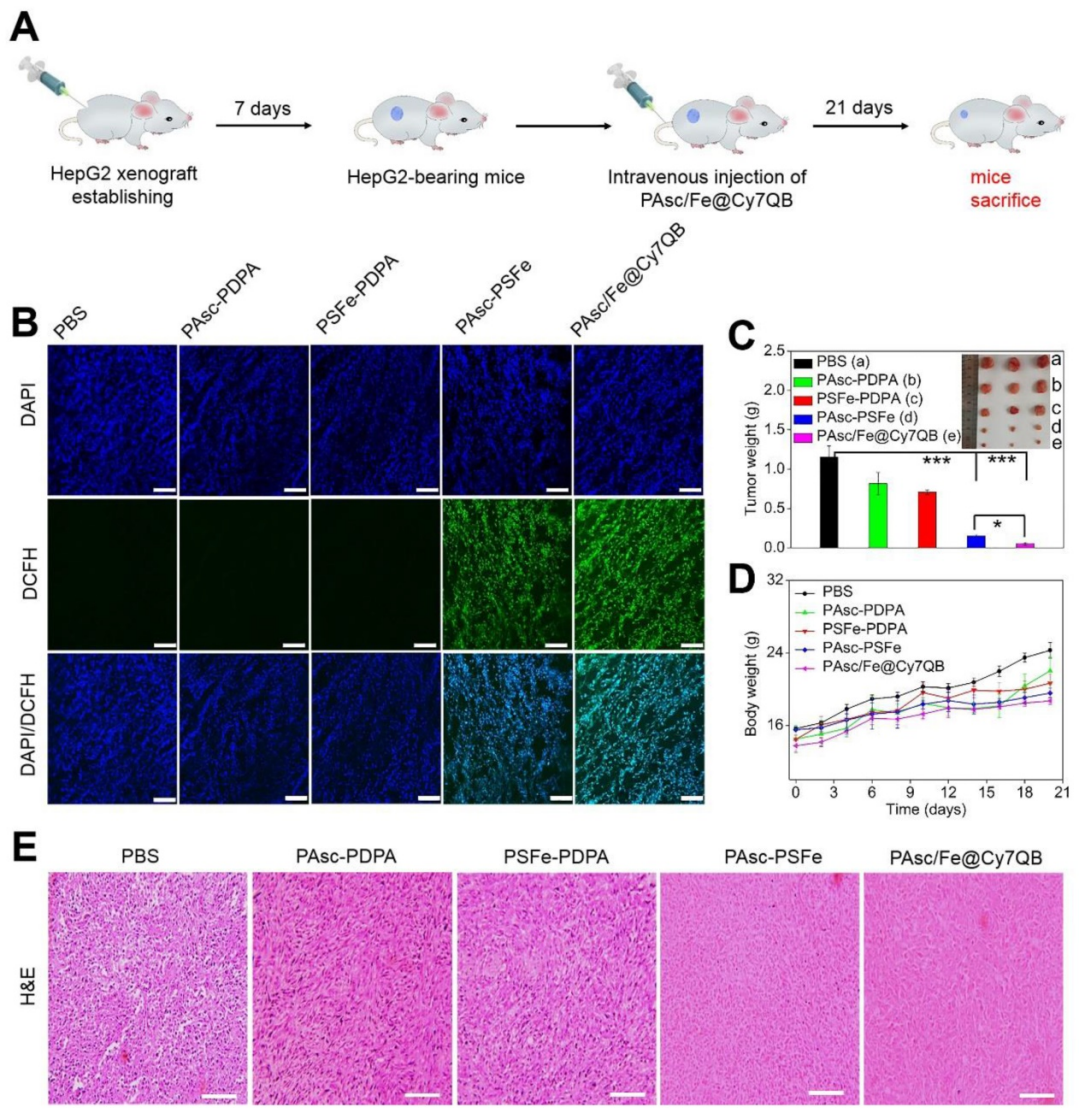
* In vivo* antitumor efficacy against HepG2 tumors of intravenous injection of PBS, PAsc-PDPA, PSFe-PDPA, PAsc-PSFe, or PAsc/Fe@Cy7QB. (A) Schematic illustration of the establishment of the HepG2 tumor xenograft model and treatment process. (B) DCFH-staining of tumor tissues from different groups at 24 h post-injection. (C) Typical images and the relative tumor weight of HepG2 tumor-bearing mice in various groups after 21 days of treatment. (D) Bodyweight changes of HepG2 tumor-bearing mice in various groups after 21 days of treatment. (E) H&E-stained slices of tumor tissues from different groups collected after 21 days of treatment. Mean ± s.d, n = 3. Scale bar represents 20 μm. P values were calculated by two-tailed Student's t-test (***p < 0.001, or *p < 0.05).

Based on the H_2_O_2_-catalytic substantial production of •OH and remarkable tumor-selective imaging *in vitro* and *in vivo*, PAsc/Fe@Cy7QB were injected into HepG2-bearing mice to investigate its anticancer efficacy (Figure 6A). The ROS levels in tumor tissues of treated mice were detected using the DCFH-DA probe. As shown in Figure 6B, bright green fluorescence was observed in the group that was injected with PAsc-PSFe due to the self-catalytic Fenton reaction. However, the group treated with PAsc/Fe@Cy7QB presented brighter imaging due to the combination of self-catalytic Fenton reaction and GSH elimination. To verify the therapeutic efficacy of the nanomicelles, mice bearing HepG2 tumors with initial volumes of 100-150 mm^3^ were chosen and randomly divided into five groups (n = 3 per group). One group was injected with saline and used as a control and the other four groups received PAsc-PDPA, PSFe-PDPA, PAsc-PSFe, or PAsc/Fe@Cy7QB i.v. injections and were then subjected to the indicated treatments. Mice in control, PAsc-PDPA and PSFe-PDPA groups exhibited rapid tumor growth, indicating that these agents had almost no therapeutic effect. The PAsc-PSFe group inhibited tumor growth due to the self-catalytic Fenton reaction, whereas the PAsc/Fe@Cy7QB group exhibited the best tumor suppression effect, demonstrating the efficient and superior anticancer effect of the micelles (Figure 6C, S34). H&E staining was subsequently performed to evaluate the treatment effect, and the results demonstrated more necrosis and karyolysis in the PAsc/Fe@Cy7QB group (Figure 6E). No obvious loss in body weight was recorded in any of the experimental groups (Figure 6D), and the hemolysis test reflected the biosafety of PAsc/Fe@Cy7QB even at high concentrations (Figure S35). H&E images of major organs in all treated groups revealed no abnormality or organ toxicity compared to the control group, indicating barely any side effects and good biocompatibility of PAsc/Fe@Cy7QB (Figure S36). Therefore, the high biodegradability and biocompatibility with the efficient tumor-targeted pharmacological effects suggested great potential of PAsc/Fe@Cy7QB for precise cancer therapy.

## Conclusion

We have successfully developed a novel *in situ* activatable PAsc/Fe@Cy7QB nanomicelles for the tumor-targeted chemodynamic theranostics to achieve effective eradication of localized solid tumors. The biodegradable nanomicelles featured optimized nanoparticle size, PEG surface, and high stability under physiological conditions and could persist for prolonged periods of times in the blood circulation to improve tumor accumulation. The tumor microenvironmental acidosis facilitated the selective disassembly of PAsc/Fe@Cy7QB, inducing the release of Fe^2+^, AscH^-^ and the adjuvant Cy7QB. The released pharmaceutical components synergized to achieve the tumor-cell recognition with multimodal imaging as well as the burst cascade of •OH to selectively kill tumor cells. Our study provides a new paradigm for Fenton metal-based chemodynamic theranostics, demonstrating great potential for accurate tumor therapies.

## Supplementary Material

Supplementary figures and table, experimental section.

## Abbreviations

Asc: ascorbate (ascorbic acid)
AscH^-^: ascorbate monoanion
Asc^·-^: ascorbate radical
Cy7QB: Cy7 dye incorporated with quinone methide-generating boronate ester
PDPA: poly(2-(diisorpropylamino) ethyl methacrylate)
PAsc-PSFe: polymerized ascorbate, Schiff base-Fe^2+^ complex and 2-(diisorpropylamino) ethyl methacrylate
PAsc-PDPA: polymerized ascorbate and 2-(diisorpropylamino) ethyl methacrylate
PSFe-PDPA: polymerized Schiff base-Fe^2+^ complex and 2-(diisorpropylamino) ethyl methacrylate
CDT: chemodynamic therapy
•OH: hydroxyl radicals
H_2_O_2_: hydrogen peroxide
GSH: glutathione
EPR: enhanced permeability and retention
EDS: energy dispersive spectrometer
MRI: magnetic resonance imaging
FLI: fluorescence imaging
PAI: photoacoustic imaging
ESR: electron spin resonance
BA: benzoic acid
TEM: transmission electron microscopy
DLS: dynamic light scattering
TMB: 3,3′,5,5′-tetramethylbenzidine
HRP: horseradish peroxidase
PMA: phorbol 1,2-myristate 1,3-acetate
CAT: Catalase
DMEM: Dulbecco's modified Eagle's medium
CLSM: confocal fluorescence microscope images
DCFH-DA: 2,7-dichlorofluorescein diacetate
MTT: 3-(4,5-dimethylthiazol-2-yl)-2,5-diphenyltetrazolium bromide
FITC: 4-nitrophthalonitrile fluorescein isothio-cyanate
PI: propidium iodide
RES: reticuloendothelial system
H&E: hematoxylin and eosin
HepG2: human hepatoma cells
