# Supplementary material for: Eradication of solid tumors by chemodynamic theranostics with H2O2-catalyzed hydroxyl radical burst
Source: Theranostics. 2021 Jan 1;11(5):2334–48. doi: 10.7150/thno.49277 (PMC7797687; doi:10.7150/thno.49277)
Supplement: Supplementary file 1 — Supplementary figures and table, experimental section. [file thnov11p2334s1.pdf]

Supplementary material

Eradication of solid tumors by chemodynamic theranostics with H_2O_2-catalyzed hydroxyl radical burst

Nana Wang,[1,2] Qin Zeng,[1,2] Ruijing Zhang,[1,2] Da Xing*[1,2] and Tao Zhang*[1,2]

[1]MOE Key Laboratory of Laser Life Science & Institute of Laser Life Science, College of Biophotonics, South China Normal University, Guangzhou, 510631, P.R. China

[2]Guangdong Provincial Key Laboratory of Laser Life Science, College of Biophotonics, South China Normal University, Guangzhou, 510631, P.R. China

E-mail: xingda@scnu.edu.cn, zt@scnu.edu.cn

$$Fe^{2+} + O_2 \longrightarrow Fe^{3+} + O_2^{\bullet -} \quad (1)$$

$$Fe^{3+} + AscH^{-} \longrightarrow Fe^{2+} + Asc^{\bullet -} \quad (2)$$

$$2O_2^{\bullet -} + 2H^{+} \longrightarrow O_2 + H_2O_2 \quad (3)$$

$$Fe^{2+} + H_2O_2 \longrightarrow Fe^{3+} + {\bullet}OH \quad \text{Fenton reaction} \quad (4)$$

Figure S1. The mechanism for generation of hydroxyl radicals (•OH).

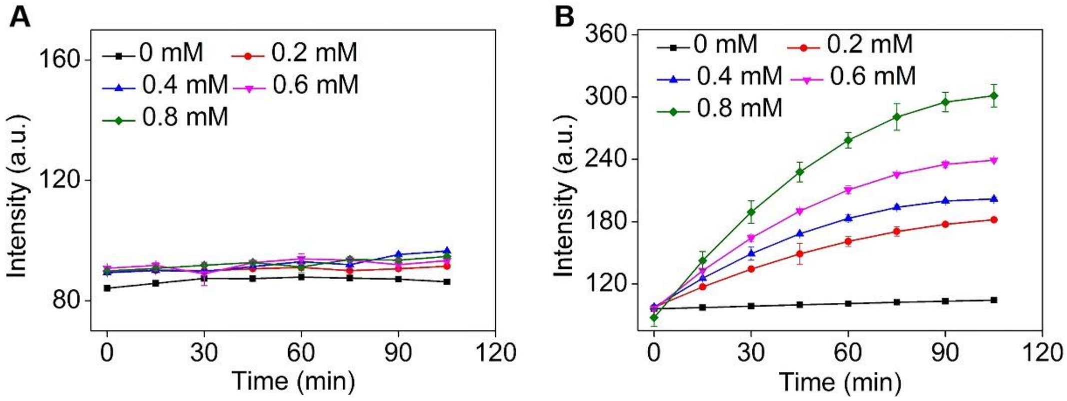

Figure S2. Effect of ascorbate concentration on the generation of •OH *via* Fenton reaction. Fluorescence intensity of BA in the presence of free Fe^{2+} (0.16 mM) and ascorbate (0, 0.1, 0.2, 0.4, 0.6, and 0.8 mM) at (A) pH 7.4 and (B) pH 5.0.

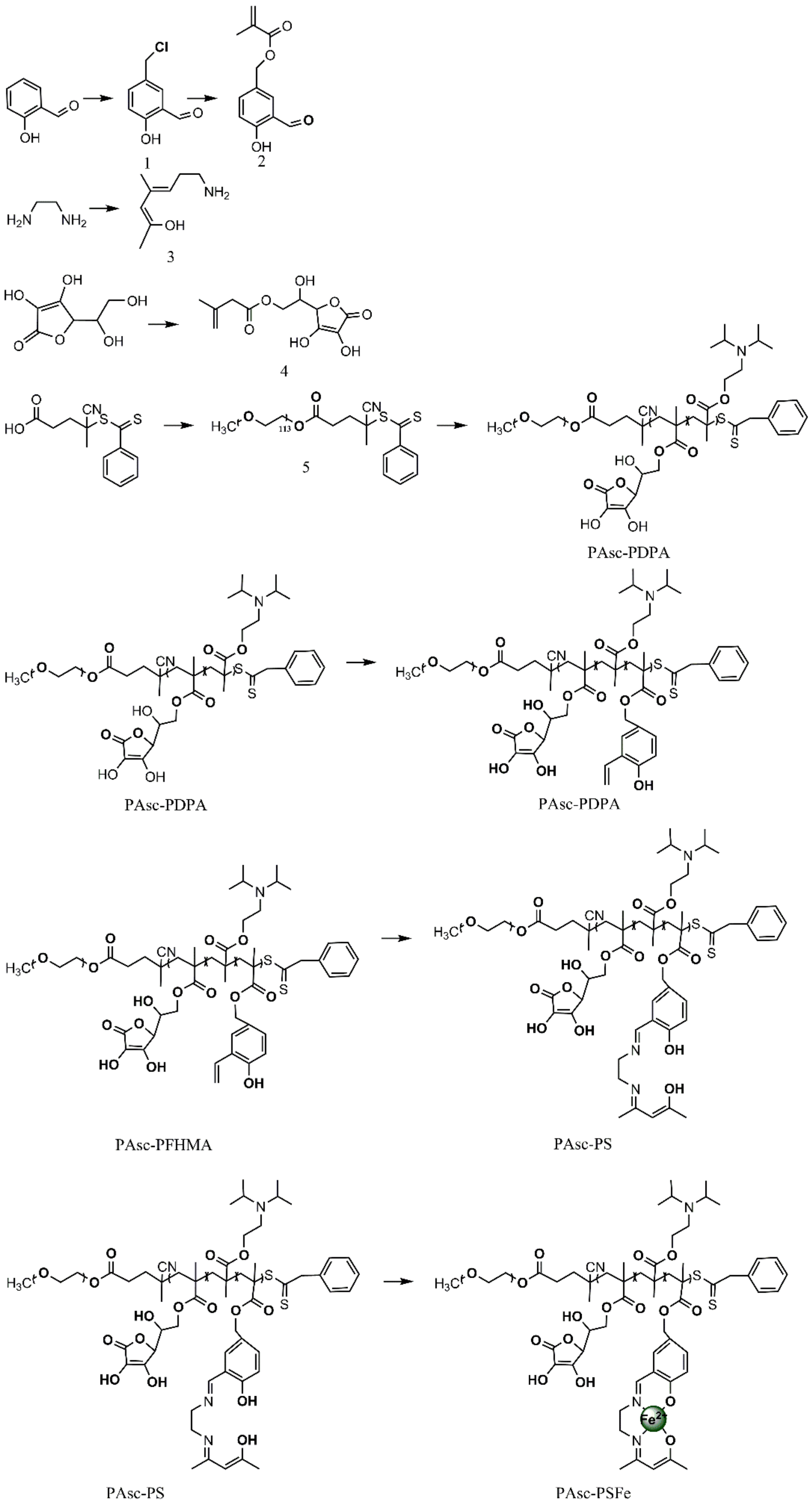

Figure S3. Synthetic route of PAsc-PSFe.

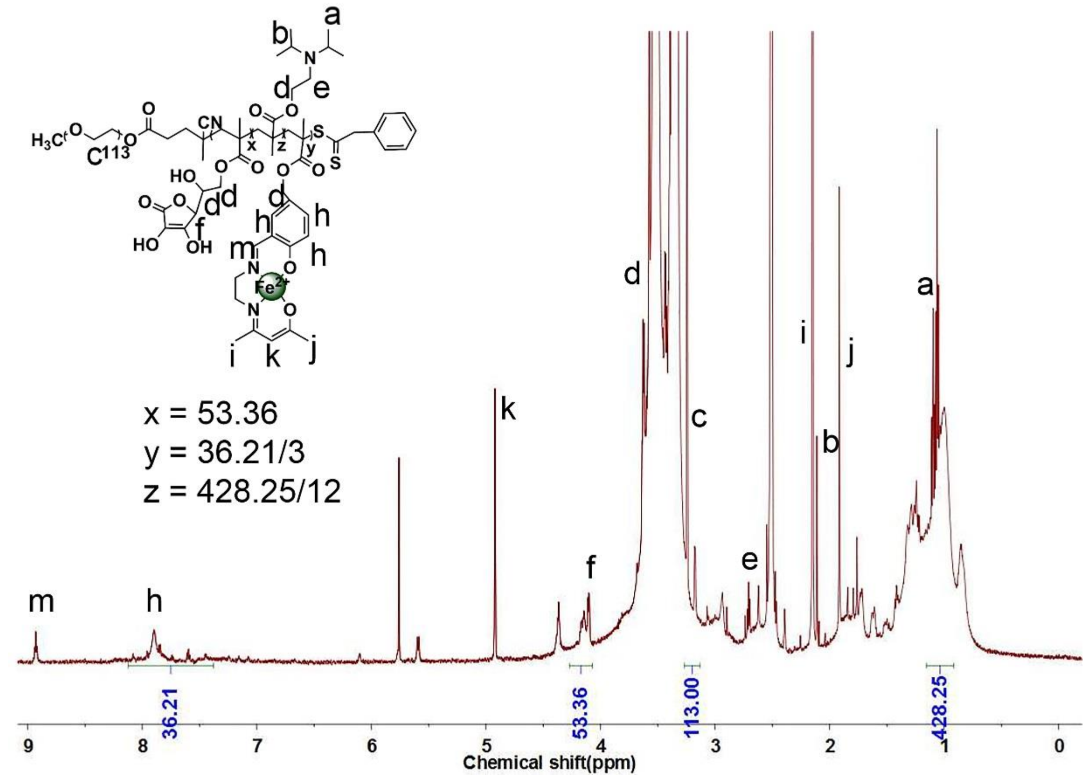

Figure S4. ^{1}H NMR spectrum of $PDPA_{36}$-b-$(PAsc_{0.82}$-$PSFe_{0.18})_{65}$ in D_6-DMSO.

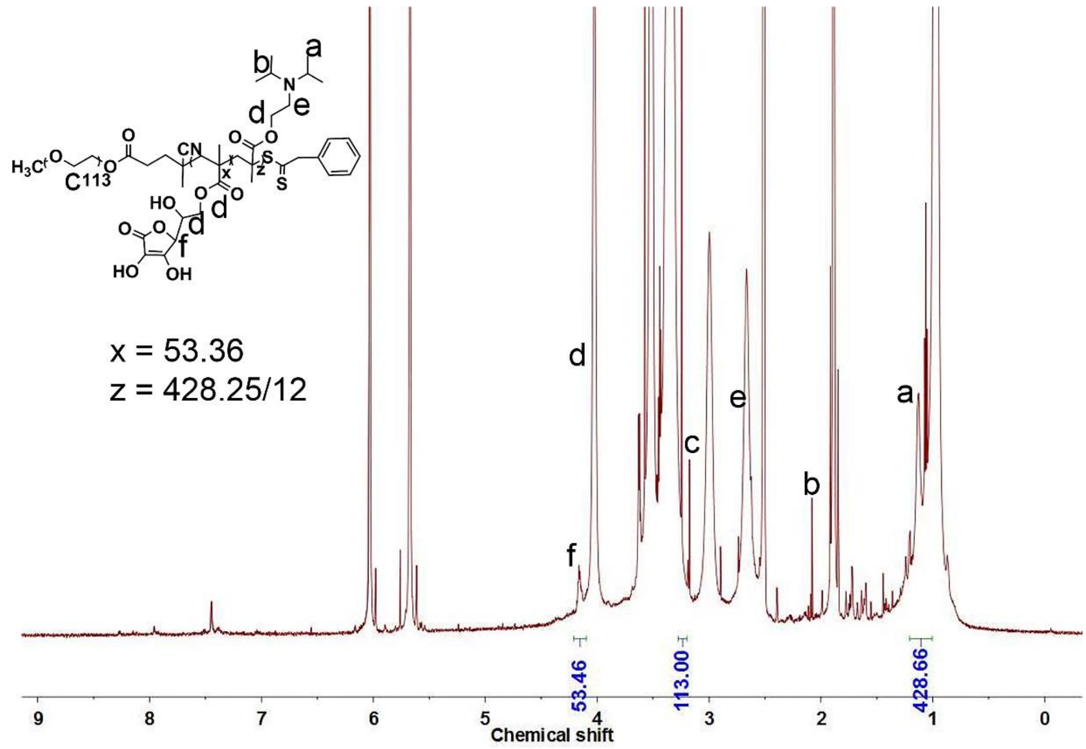

Figure S5. ^{1}H NMR spectrum of $PAsc_{53}$-$PDPA_{36}$ in d^6-DMSO.

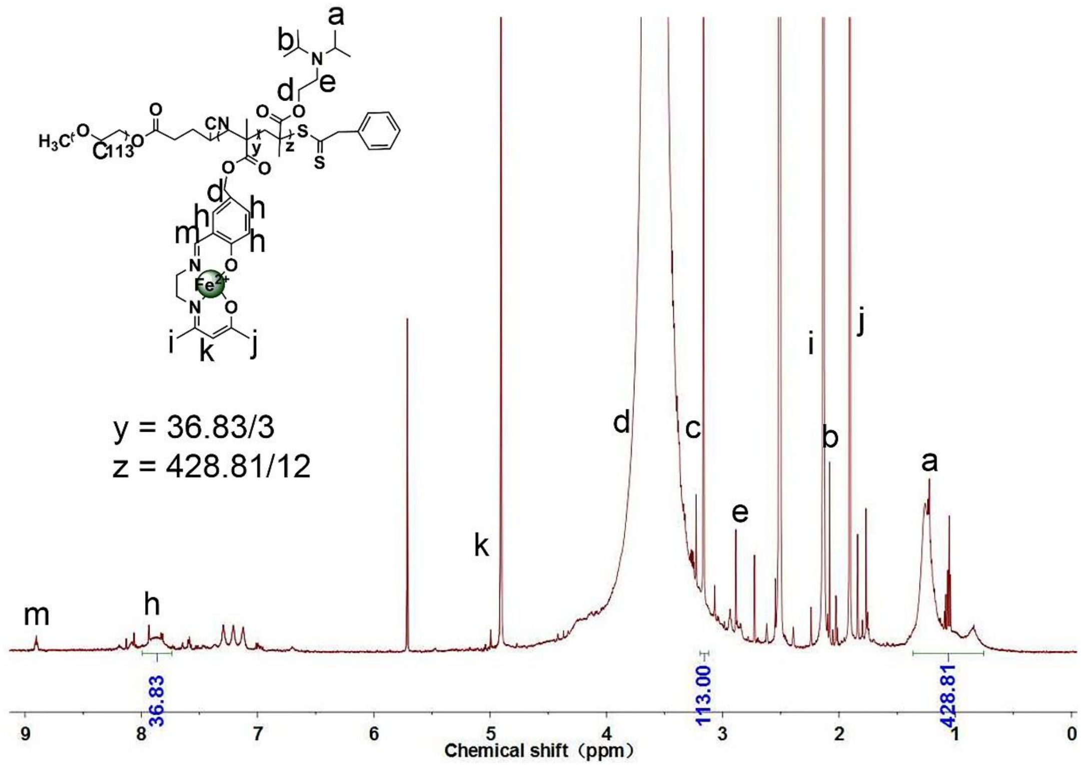

Figure S6. ^{1}H NMR spectrum of $PSFe_{12}$-$PDPA_{36}$ in d^6-DMSO.

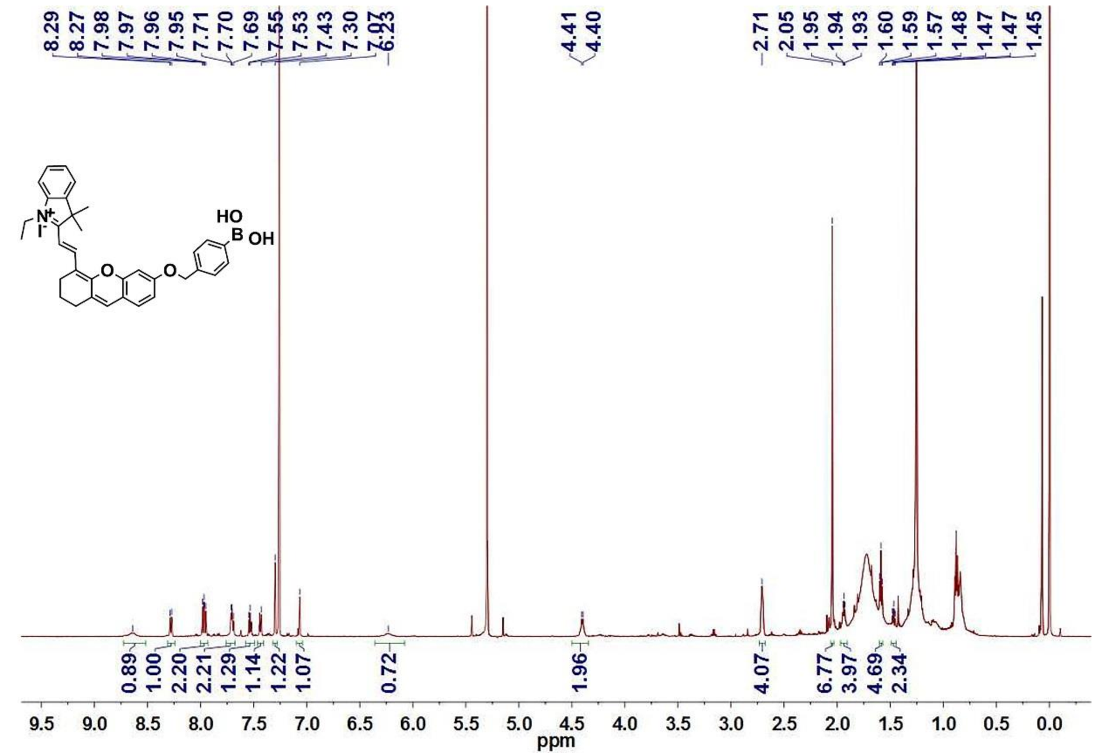

Figure S7. ^{1}H NMR spectrum of Cy7QB in $CDCl_3$. ^{1}H NMR (600 MHz, $CDCl_3$, ppm): δ 8.29 (brs, 1H), 8.27 (d, J = 12 Hz, 1H), 7.95 (m, 2H), 7.69 (t, J = 6.0 Hz, 2H), 7.52 (t, J = 6.0 Hz, 1H), 7.43

(d, J = 12 Hz, 1H), 7.30 (s, 1H), 6.23 (brs, 1H), 4.40 (d, J = 6.0 Hz, 2H), 2.71 (s, 4H), 2.05 (s, 6H), 1.93 (t, J = 6.0 Hz, 4H), 1.57 (t, J = 6.0 Hz, 3H), 1.45 (m, 2H).

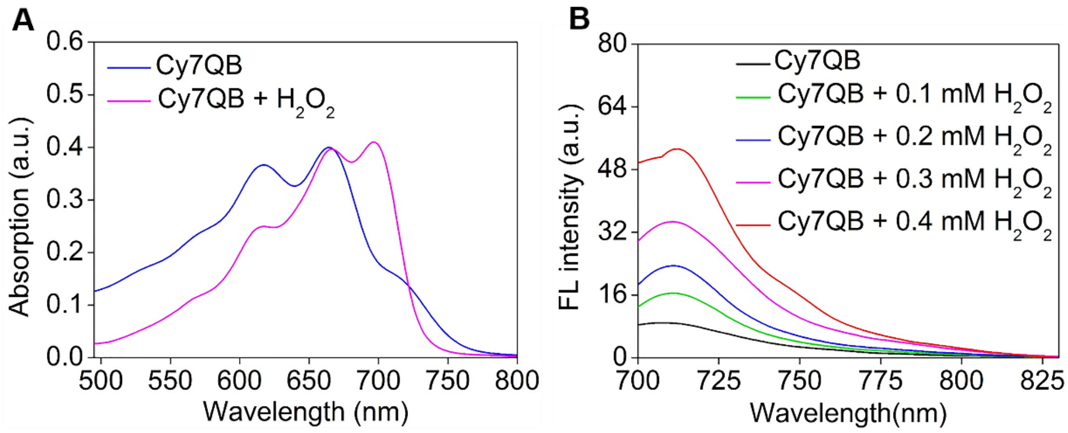

Figure S8. Absorption (A) and fluorescence (B) spectra of Cy7QB (λ_{ex} = 680 nm) under H_2O_2 treatments.

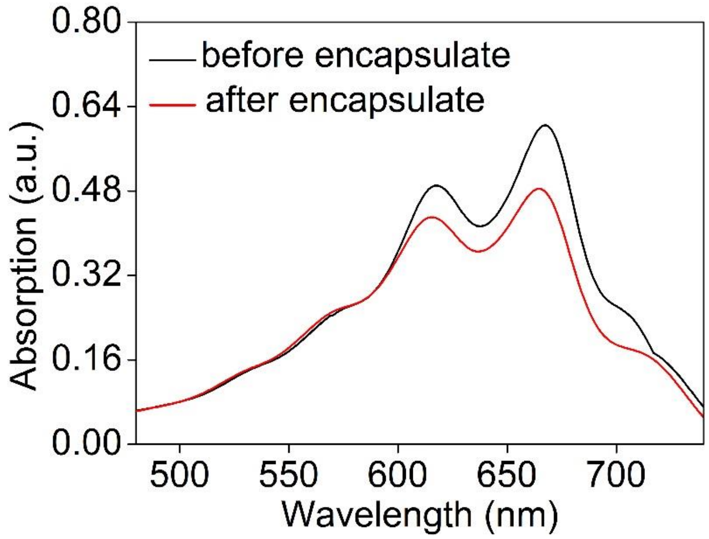

Figure S9. Encapsulation efficiency of Cy7QB within the nanoparticles PAsc/Fe@Cy7QB.

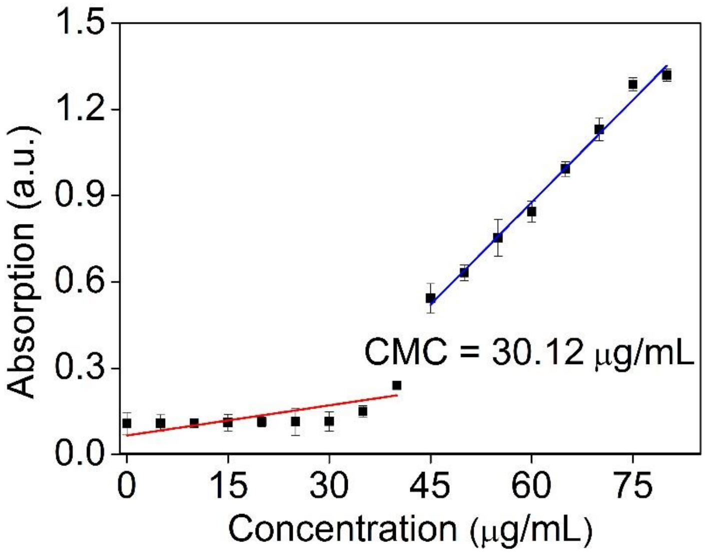

Figure S10. Critical micelle concentration (CMC) of PAsc/Fe@Cy7QB.

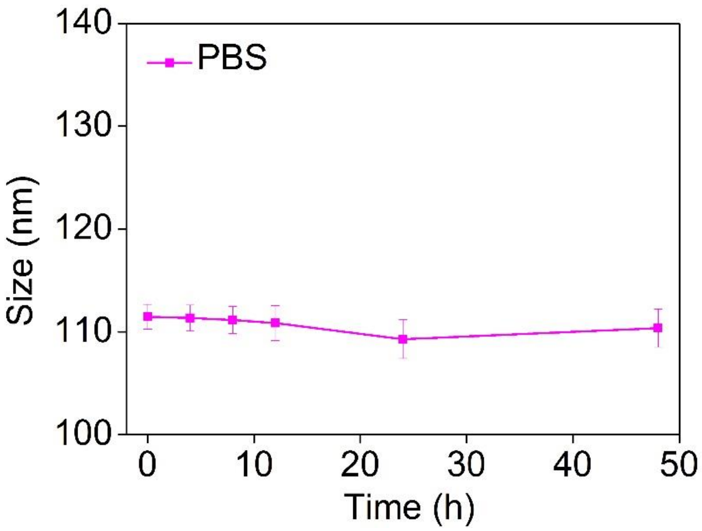

Figure S11. Hydrodynamic diameter of PAsc/Fe@Cy7QB in PBS at room temperature during 48-hour observation.

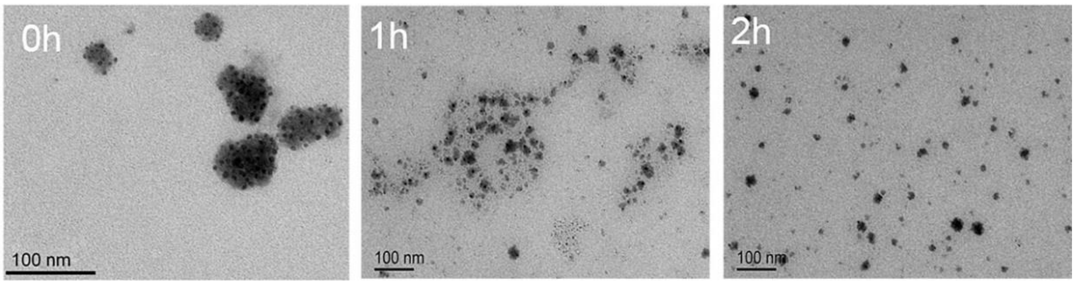

Figure S12. TEM image of PAsc/Fe@Cy7QB by dispersing in pH 5.0 with different time (0, 1, 2 h).

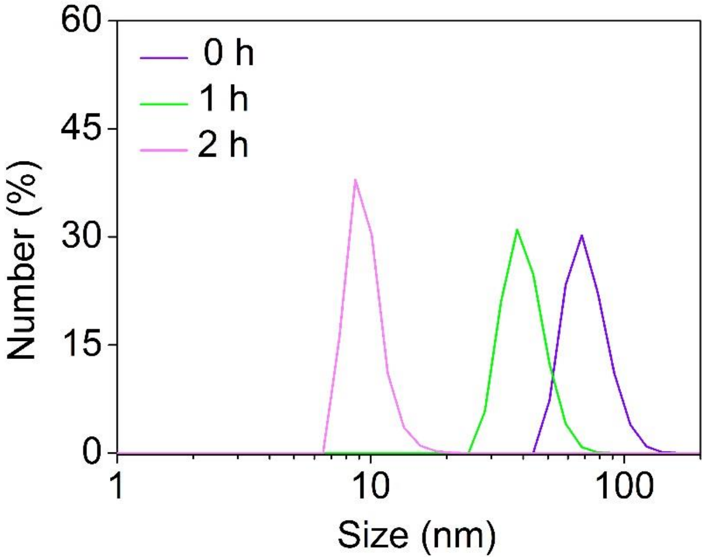

Figure S13. DLS spectra of PAsc/Fe@Cy7QB dispersing in pH 5.0 with different time.

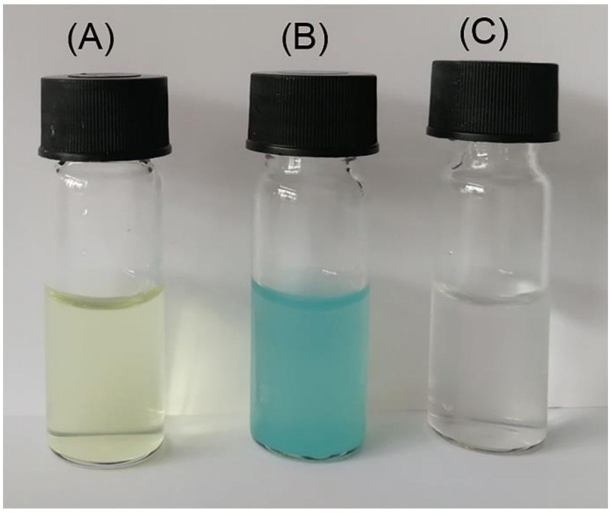

Figure S14. Photo of PAsc-PSFe solution in the presence of potassium ferricyanide at pH 7.4 (A) and pH 5.0 (B), or in the presence of potassium thiocyanate at pH 5.0 (C) for 24 h.

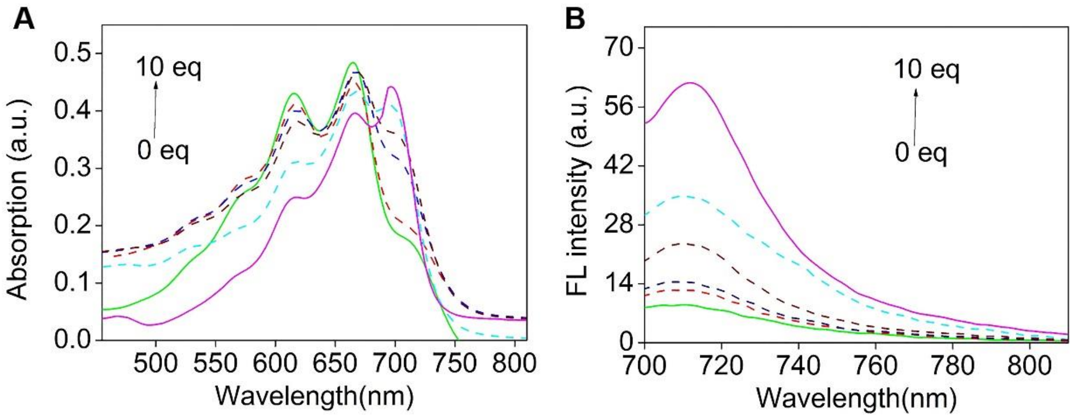

Figure S15. Absorption (A) and fluorescence (B) spectra of PAsc/Fe@Cy7QB (50 μg/mL, pH 5.0) PBS toward varied concentrations of H_2O_2 (0 - 10 eq).

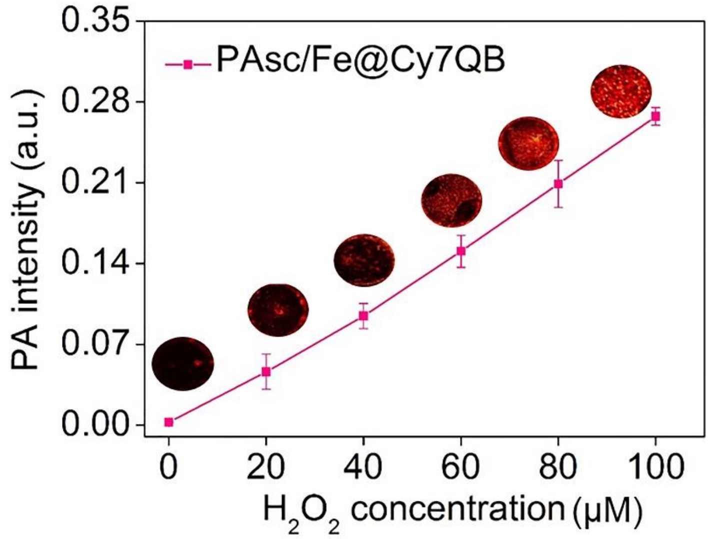

Figure S16. PA images and PA signal intensities of PAsc/Fe@Cy7QB at 670 nm with different H_2O_2 concentrations.

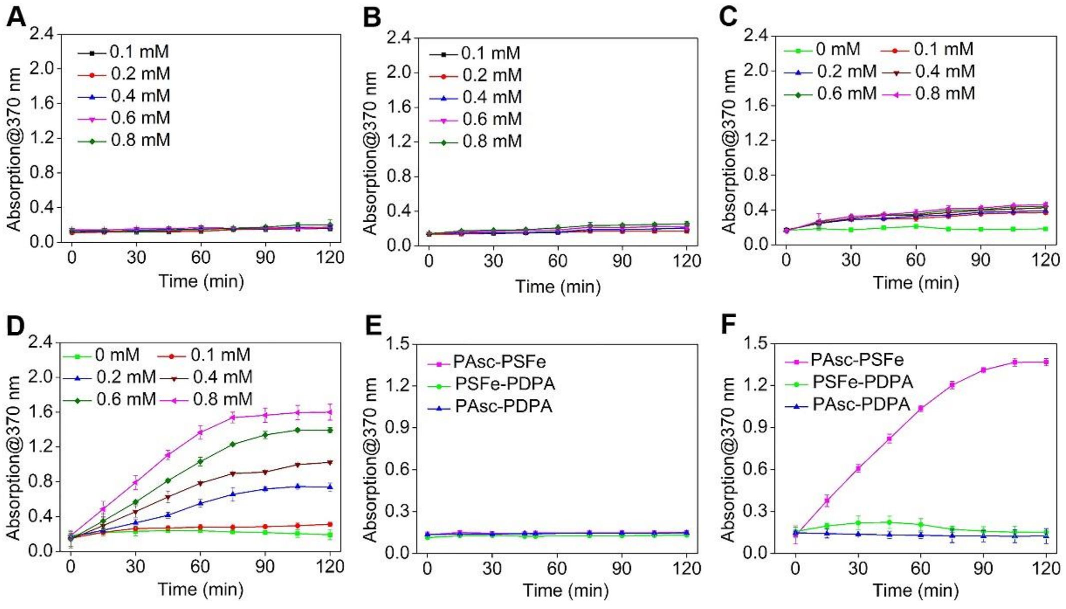

Figure S17. Time-dependent absorbance of oxidized TMB in the presence of HRP (150 mU/mL), and TMB (100 μM) after treatment with ascorbate (0, 0.1, 0.2, 0.4, 0.6, and 0.8 mM) at (A) pH 7.4 and (B) pH 5.0. Treatment with Fe^{2+} (0.16 mM) and ascorbate (0, 0.1, 0.2, 0.4, 0.6, and 0.8 mM) at (C) pH 7.4 and (D) pH 5.0. Treatment with PAsc-PSFe, PAsc-PDPA, or PSFe-PDPA at (E) pH

7.4 and (F) pH 5.0.

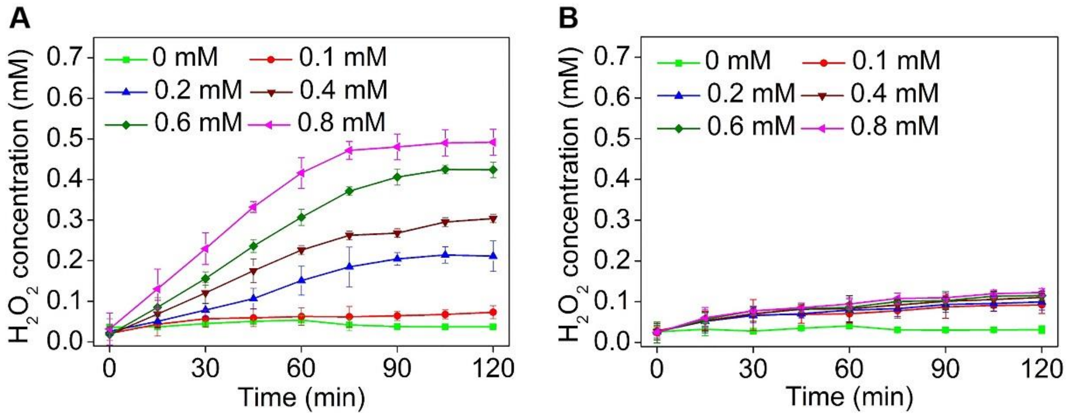

Figure S18. Effect of ascorbate concentration on the generation of H_2O_2. Time-dependent production of H_2O_2 with the coexistence of free Fe^{2+} (0.16 mM) and ascorbate (0, 0.1, 0.2, 0.4, 0.6, and 0.8 mM) at **(**A) pH 5.0 and (B) pH 7.4.

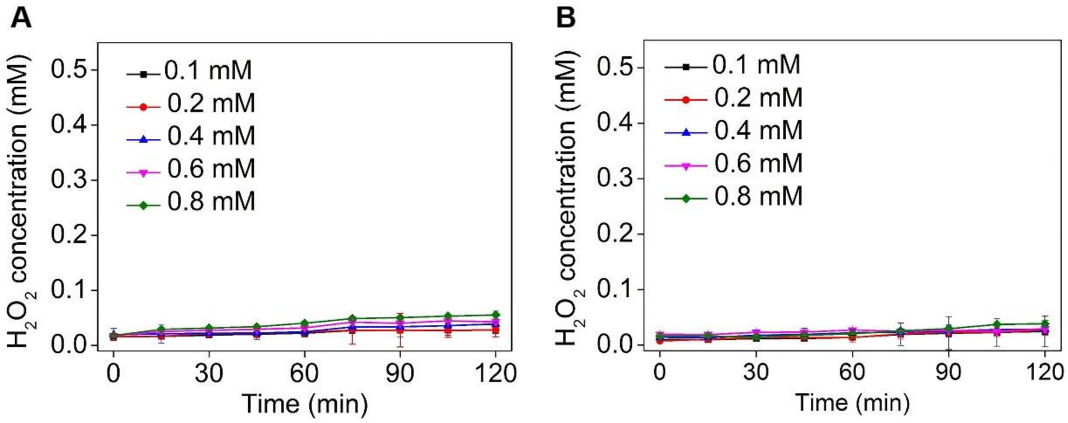

Figure S19. Effect of ascorbate concentration on the generation of H_2O_2. Time-dependent production of H_2O_2 after treatment with ascorbate (0.1, 0.2, 0.4, 0.6, and 0.8 mM) at (A) pH 5.0 and (B) pH 7.4.

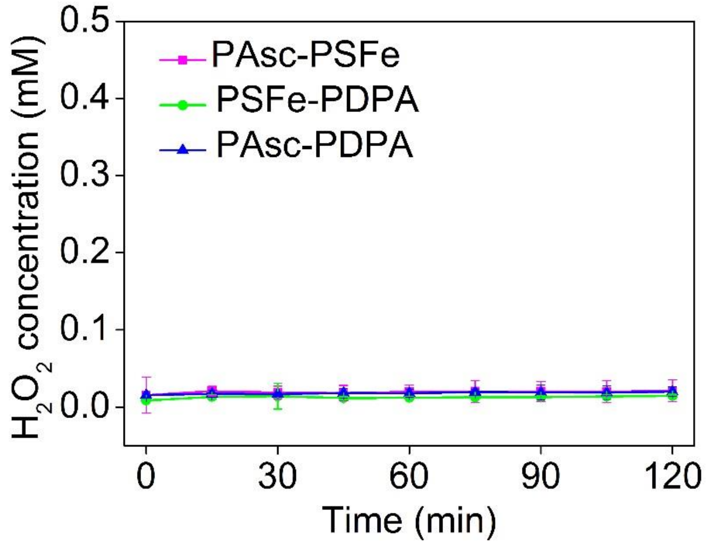

Figure S20. Time-dependent production of H_2O_2 after treatment with at PAsc-PSFe, PAsc-PDPA or PSFe-PDPA at pH 7.4.

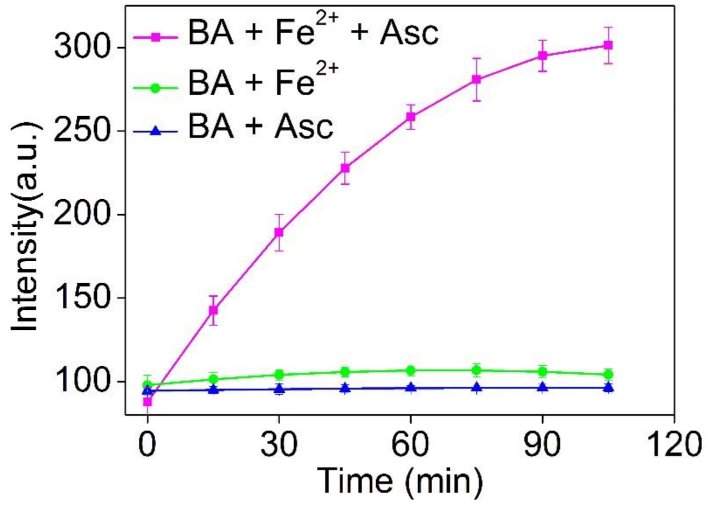

Figure S21. Time-dependent detection of •OH after treatment with ascorbate (0.8 mM) and Fe^{2+} (0.16 mM), Fe^{2+} (0.16 mM) or ascorbate (0.8 mM) at pH 5.0.

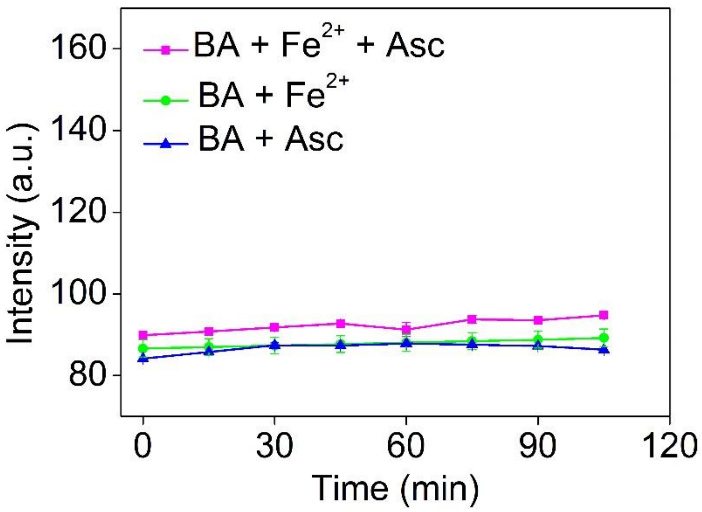

Figure S22. Time-dependent detection of •OH after treatment with ascorbate (0.8 mM) and Fe^{2+} (0.16 mM), Fe^{2+} (0.16 mM) or ascorbate (0.8 mM) at pH 7.4.

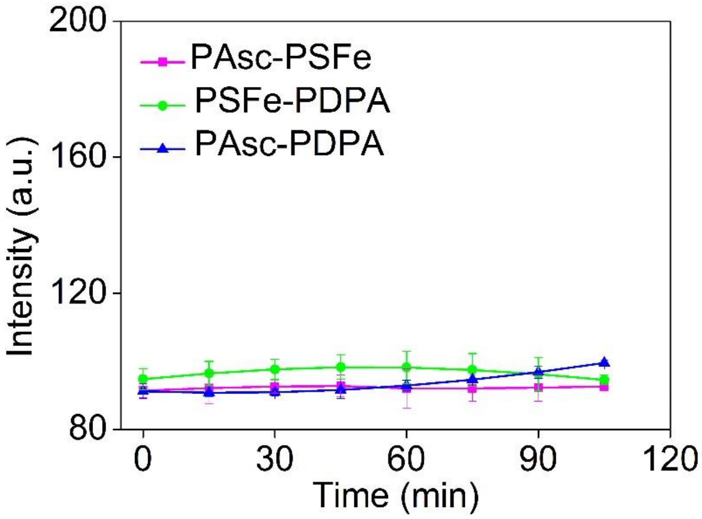

Figure S23. Time-dependent detection of •OH after treatment with PAsc-PSFe, PAsc-PDPA, or PSFe-PDPA at pH 7.4.

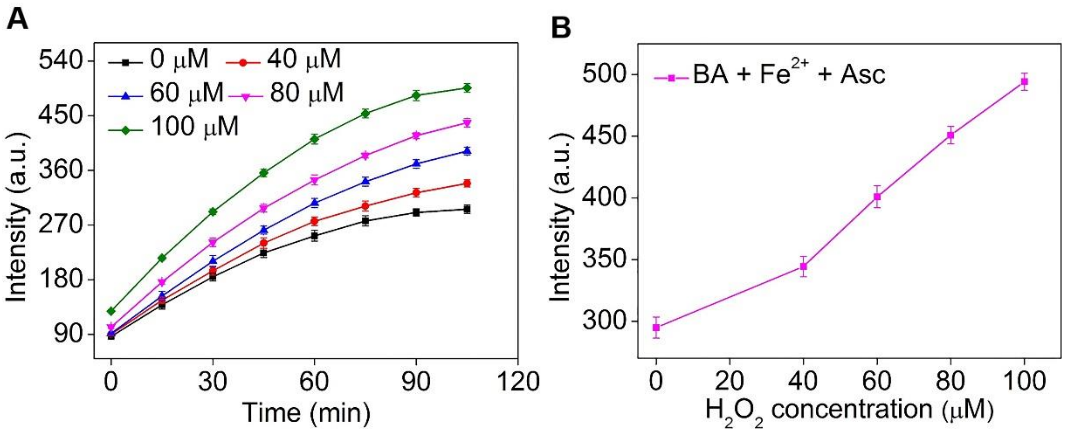

Figure S24. Time-dependent detection of •OH at different H_2O_2 concentrations. Samples treated with ascorbate (0.8 mM) and Fe^{2+} (0.16 mM).

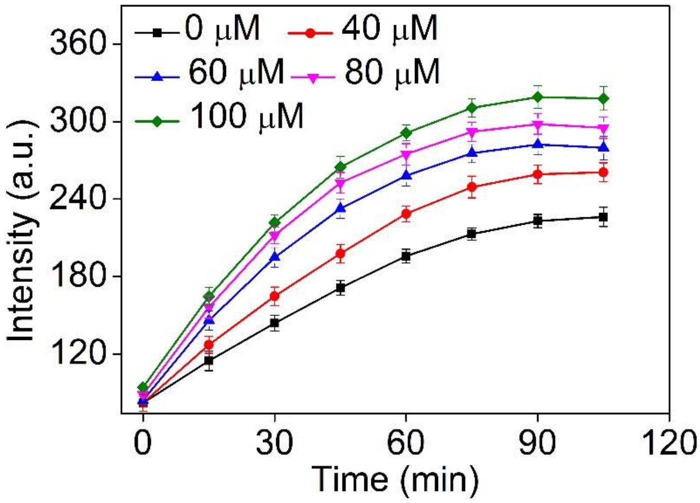

Figure S25. Time-dependent detection of •OH at different H_2O_2 concentrations. Polymers treated with PAsc-PSFe.

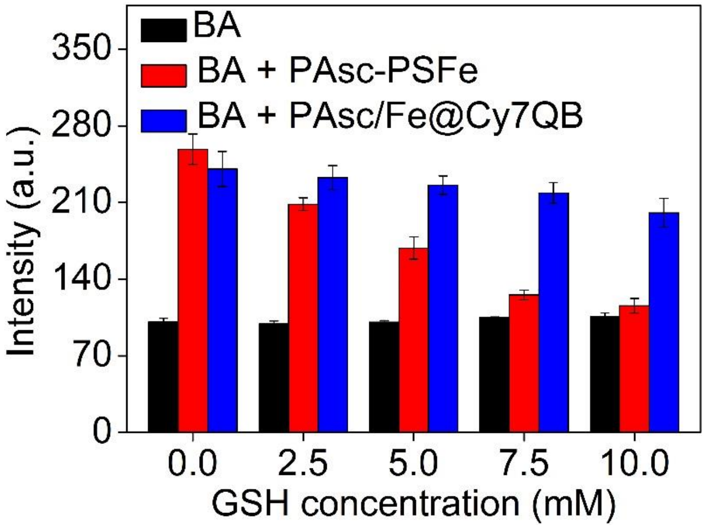

Figure S26. Fluorescence spectra of BA by •OH generated by different concentration of GSH-treated PAsc-PSFe or PAsc/Fe@Cy7QB respectively at pH 5.0.

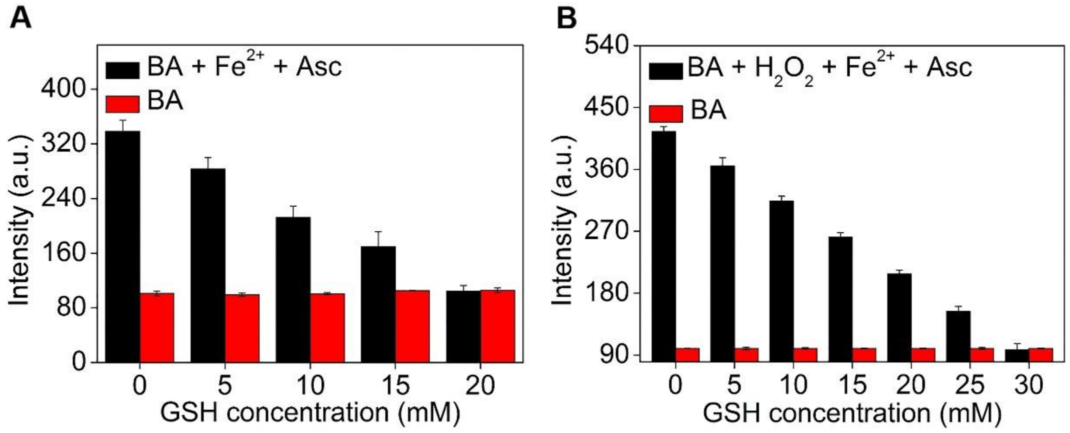

Figure S27. Detection of •OH by different concentration of GSH-treated ascorbate (0.8 mM) + Fe^{2+} (0.16 mM) or Fe^{2+} (0.16 mM) + ascorbate (0.8 mM) + H_2O_2 (100 μM).

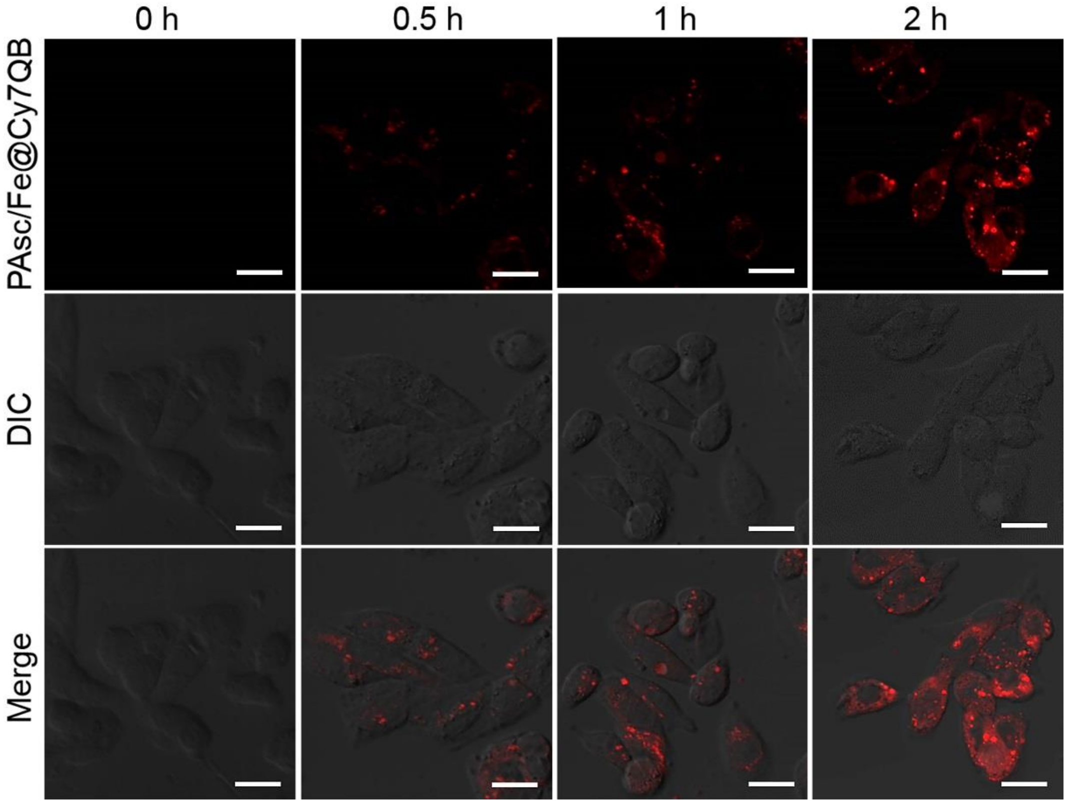

Figure S28. Microscopic images of the time-dependent fluorescence in the HepG2 cells incubated with PAsc/Fe@Cy7QB (50 μg/mL). Scale bar represents 20 μm.

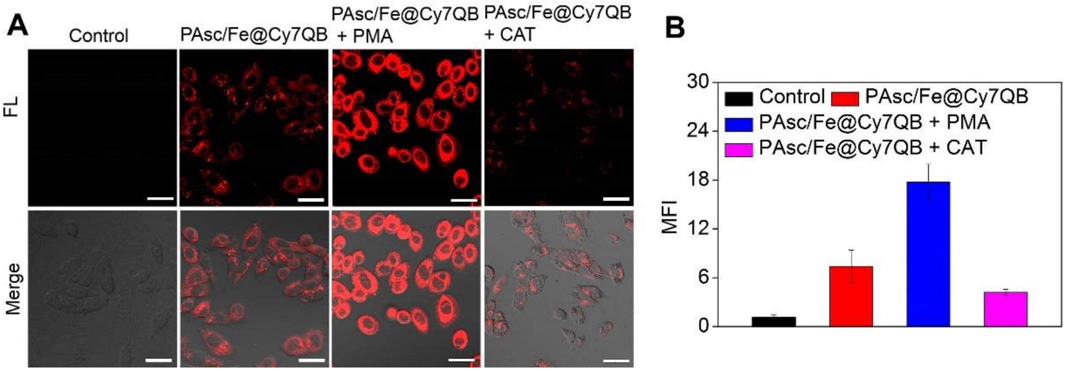

Figure S29. (A) HepG2 cells incubated with PAsc/Fe@Cy7QB on the endogenous H_2O_2. (B) The corresponding mean fluorescence intensity (MFI) values. Scale bar represents 20 μm.

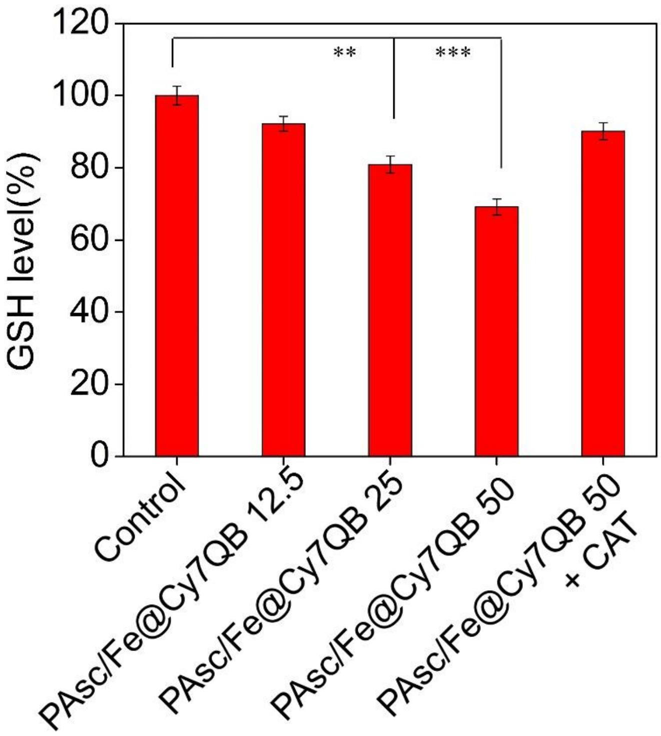

Figure S30. GSH levels in HepG2 cells treated with PAsc/Fe@Cy7QB at various concentrations (12.5, 25, or 50 μg/mL) or treated with PAsc/Fe@Cy7QB (50 μg/mL) and CAT. P values were calculated by two-tailed Student's t-test (***$p < 0.001$, or *$p < 0.05$).

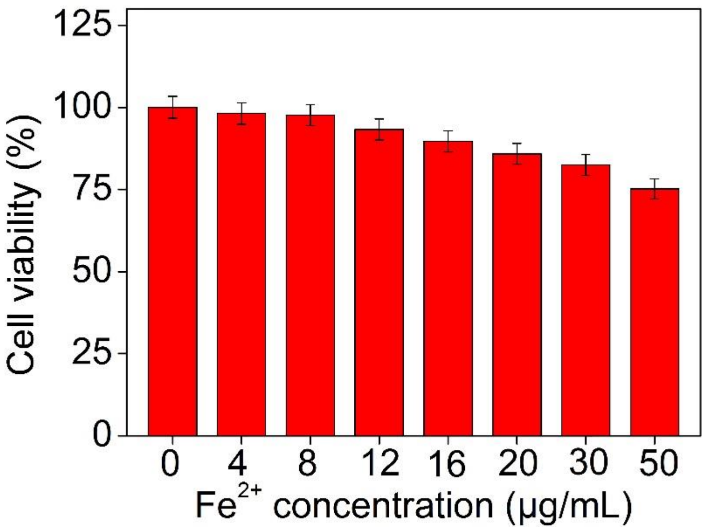

Figure S31. The cell viability of HepG2 cells after treatment with $FeCl_2$ for 24 h at various concentrations.

Combinationa	CI at IC_{50}	Interpretation
PAsc-PSFe	0.51	Synergism
PAsc/Fe@Cy7QB	0.47	Synergism

Table S1. Combination Index (CI) values of the interaction. Combination index (CI) = 1 indicates an additive effect, CI < 1 indicates a synergistic effect and CI > 1 indicates antagonism.

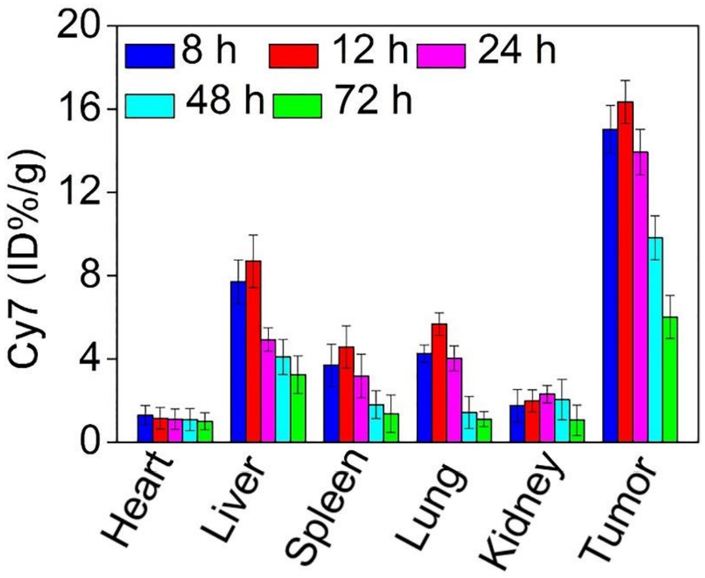

Figure S32. Biodistributions in mice bearing HepG2 tumors after intravenous administration of PAsc/Fe@Cy7QB for 8, 12, 24, 48 and 72 h.

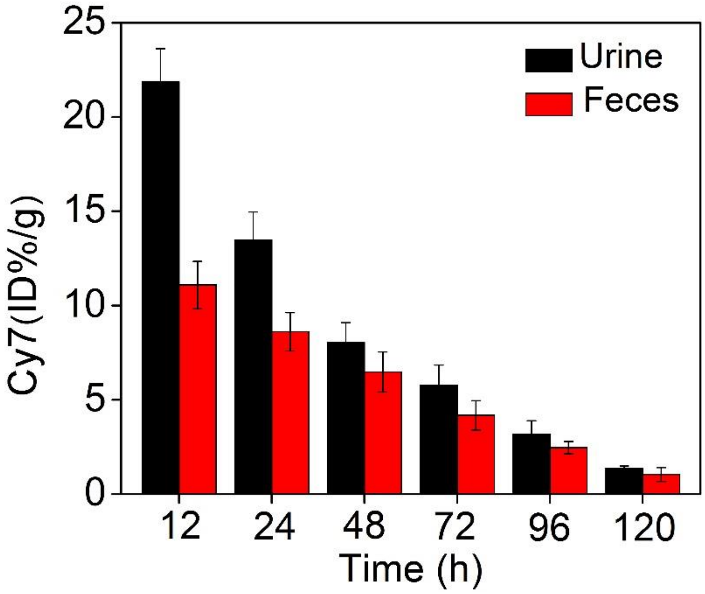

Figure S33. Excretion profile of PAsc/Fe@Cy7QB.

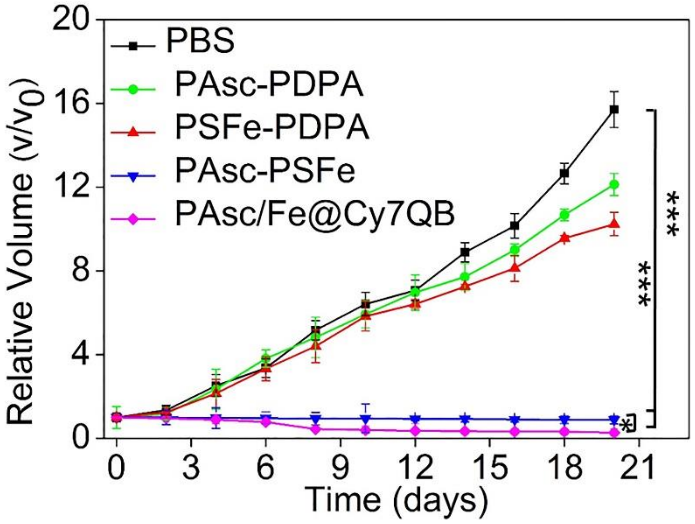

Figure S34. The relative tumor volume changes of HepG2 tumor-bearing mice in various groups after 21 days of treatment. Mean ± s.d, n = 3. P values were calculated by two-tailed Student's t-test (***p < 0.001, or *p < 0.05).

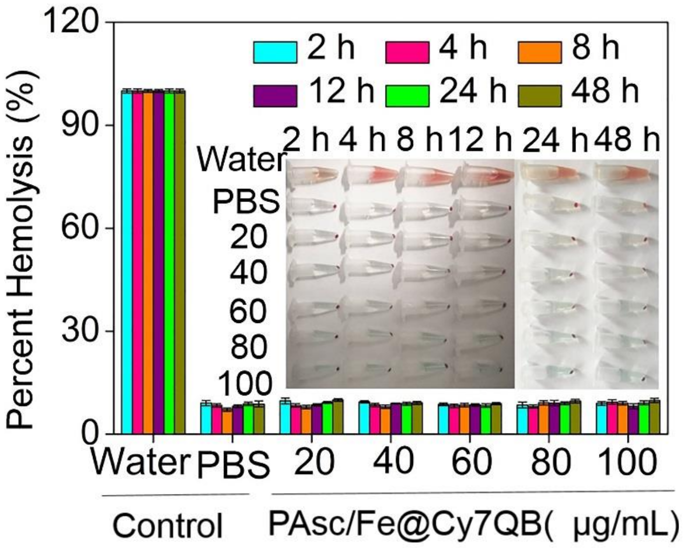

Figure S35. Hemolysis assay of PAsc/Fe@Cy7QB.

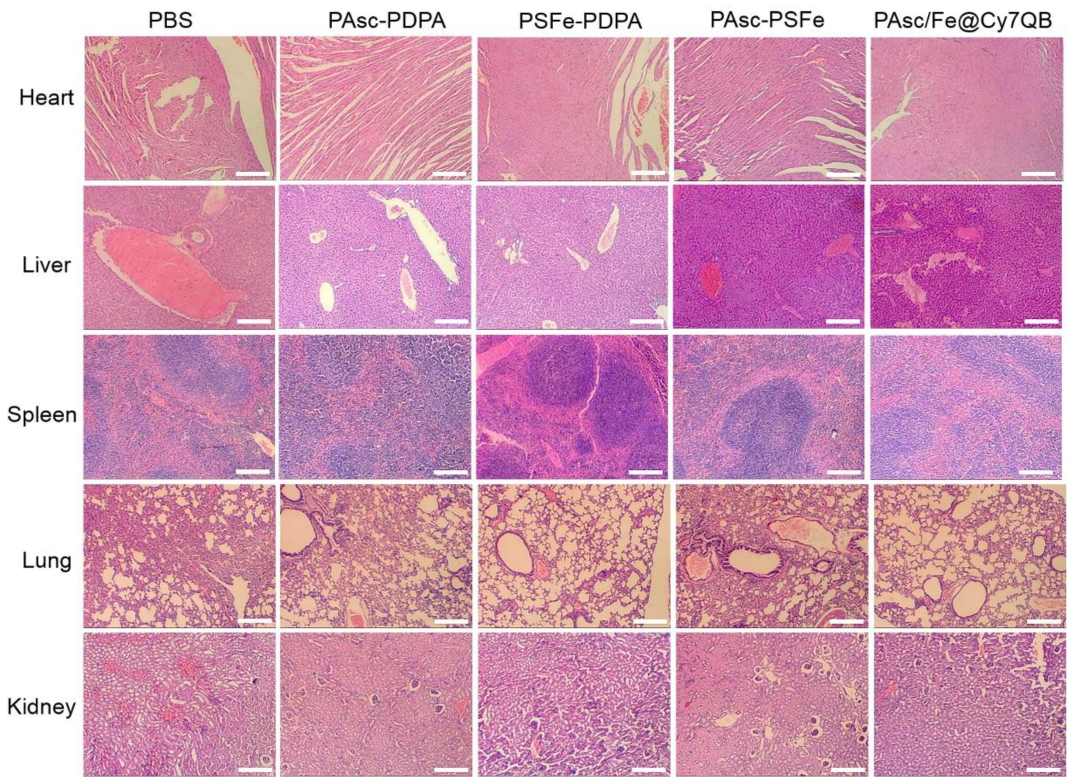

Figure S36. H&E-stained images of major organs from different treated mice. Scale bar represents 20 μm.

Experimental Section

Synthesis of compound 1 and 2

Salicylaldehyde (0.025 mol) and paraformaldehyde (0.015 mol) were added to a three-necked flask with 15 mL of concentaated. HCl. The mixture was stirred, and 1.1 mL of $POCl_3$ were gradually added dropwise within 1 h. The mixture was reacted at room temperature for 18 h. The white solid was filtered, washed with 3% $NaHCO_3$ solution and distilled water, dried and recrystallized in petroleum ether. A mixture of methacrylic acid (0.4 g, 0.005 mol), sodium hydroxide (0.2 g, 0.005 mol) and water (5 mL) was stirred at room temperature for 30 min. Compound 1 (0.85 g, 0.005 mol), potassium iodide (0.15 g, 0.0003 mol) and toluene (1 mL) were added successively to the above aqueous solution. The reaction mixture was heated to 50 °C and stirred overnight. The mixture was extracted with ethyl acetate (50 mL × 3), and the organic extract was dried over anhydrous Na_2SO_4. After filtration, the volatiles were removed by using a rotary evaporator. The residue was purified by column chromatography on silica gel with petroleum ether/dichloromethane (9:1) to obtain compound 2 as a white solid with a yield of 68.5%.

Synthesis of compound 3

A solution of 2, 4-pentanedione (1.09 mL, 10.5 mmol) in dichloromethane (8 mL) was added dropwise to a solution of 1, 2-diaminoethane (1.36 mL, 20.1 mmol) in dichloromethane (8 mL). The resulting mixture was heated under reflux for 1 h. The excess 1, 2-diaminoethane was removed from the resulting mixture by rotary evaporation under vacuum at 60 °C for 30 min. The yield was 87%.

Synthesis of compound 4

Immobilized *Candida antarctica* lipase and ascorbic acid (ascorbate, Asc) were dried under high vacuum in a desiccator with phosphorous pentoxide for 24 h prior to reaction. The reaction was an enzymatic transesterification, in which the primary hydroxyl group of ascorbic acid was regioselectively acylated by 2, 2, 2-trifluoroethyl methacrylate via the acyl enzyme complex. In a typical reaction, ascorbic acid (5.0 g, 28.4 mmol), 2,2,2-trifluoroethyl methacrylate (6.07 mL, 42.6 mmol), and C. *antarctica* lipase (3.3 g, immobilized) were stirred in 100 mL of anhydrous dioxane at 60 °C. Then, 83.0 mg of 2, 6-ditertbuty-l,4-methylphenol were added to the reaction mixture to avoid vinyl polymerization at 60 °C, functionalized poly(methyl methacrylate) and during solvent evaporation. Reactions were monitored by thin layer chromatography. The enzyme was filtered; the product was washed thoroughly with dioxane and the solvent was evaporated by rotary evaporation under reduced pressure. The yield was 90%.

Synthesis of compound 5

Typically, mPEG (10.0 g, 2 mmol), CPADB (0.57 g, 2 mmol), DMAP (0.062 g, 0.5 mmol) and DCC (1.16 g, 5.63 mmol) were dissolved in 20 mL anhydrous methylene chloride. The solution was stirred at room temperature for 48 h. After filtration of the precipitate, the solution was poured into 200 mL cold ethyl ether, yielding a pink precipitate. The pink precipitate was redissolved in methylene chloride and precipitated again in cold ethyl ether. After washing two additional times with ethyl ether, the pink product mPEG-CPAD was finally obtained, and the yield was 92%.

Synthesis of compound PAsc-PDPA

Compound 5 (150 mg), compound 4 (206.6 mg, 0.8 mmol), DPAMA (1.5 mg) and AIBN (2.0 mg) were dissolved in 2 mL 1, 4-dioxane/methanol (v/v, 3:1). The mixture was deaerated by applying three freeze-pump-thaw cycles. Afterwards, the tube was immerged in a 65 °C oil bath and stirred for 24 h. After cooling to room temperature, the mixture was precipitated in cold ethyl ether. A slight pink precipitate was collected and washed twice with ethyl ether. The product was obtained after vacuum drying for 24 h with a yield of 81%.

Synthesis of compound PAsc-PFHMA

PAsc-PDPA (150 mg), compound 2 (114.28 mg, 0.16 mmol) and AIBN (2.0 mg) were dissolved in 2 mL 1, 4-dioxane/methanol (v/v, 3:1). The mixture was deaerated by applying three freeze-pump-thaw cycles. Afterwards, the tube was immersed in a 65 °C oil bath and stirred for 24 h. After cooling to room temperature, the mixture was precipitated in cold ethyl ether. A slight yellow precipitate was collected and washed twice with ethyl ether. The product was obtained after vacuum drying for 12 h with a yield of 85%.

Synthesis of compound PAsc-PS

PAsc-PDPA (150 mg) and compound 3 (22.5 mg, 0.16 mmol) were dissolved in 2 mL of 1, 4-dioxane/methanol (v/v, 3:1). The mixture was deaerated by applying three freeze-pump-thaw cycles. Afterwards, the tube was immersed in a 50 °C oil bath and stirred for 24 h. After cooling to room temperature, the mixture was precipitated in cold ethyl ether. A slight pink precipitate was collected and washed twice with ethyl

ether. The product was obtained after vacuum drying for 12 h with a yield of 80%.

Synthesis of compound PAsc-PSFe

$FeCl_2$ (20.28 mg, 0.16 mmol) in 50 mL of ethanol was added dropwise to a solution of 150 mg PAsc-PS in 50 mL of ethanol, which was stirred in a round -bottomed flask. To avoid oxidation of Fe^{2+}, a few drops of glacial acetic acid were added. The resulting solution was magnetically stirred for 12 h under nitrogen at 40 °C, then the obtained solution was evaporated at 25-30 °C. The precipitated complex was filtered off, washed with ether, and recrystallized from ice-cold ethanol and dried in air. The optimized copolymer $PDPA_{36}$-b-($PAsc_{0.82}$-$PSFe_{0.18}$)$_{65}$ (named PAsc-PSFe) was obtained and determined by ^1H NMR in d^6-DMSO. Meanwhile, the copolymer $PAsc_{53}$-$PDPA_{36}$ (named PAsc-PDPA) with copolymerization of ascorbate and 2-(disopropylamino) ethyl methacrylate was synthesized according to the similar procedure and determined by ^1H NMR in d^6-DMSO. And copolymer $PSFe_{12}$-$PDPA_{36}$ (named PSFe-PDPA) with copolymerization of Schiff base-Fe^{2+} and 2-(disopropylamino) ethyl methacrylate was synthesized according to the similar procedure and determined by ^1H NMR in d^6-DMSO.

Preparation of PAsc/Fe@Cy7QB

The Cy7QB was synthesized according to previous similar protocol and successfully characterized by ^1H NMR. Then Cy7QB (2 mg) dissolved in CH_2Cl_2 was added to a deionized water (2 mL) solution of PAsc-PSFe (10 mg). The mixture was then stirred at 25 °C for 4 h. PAsc/Fe@Cy7QB was obtained after dialysis in a cellulose dialysis bag (MWCO 3500 Da) overnight. The resulting solution was

freeze-dried for 6 h, and the product was obtained. The dry product was re-dispersed in deionized distilled water (pH 7.4) to produce the PAsc/Fe@Cy7QB solution. The PAsc/Fe@Cy7QB solution was filtered through a 0.22 μm filter to sterilize the sample before being used in cells and mice.

pH-Dependent Fe^{2+} release

To check the valence states and release of Fe^{2+}, potassium ferricyanide and potassium thiocyanate were applied. Briefly, PAsc-PSFe was dispersed in a buffer solution with pH values of 5.0 and 7.4, and the PAsc-PSFe were concentrated into 12-well plates. Then potassium ferricyanide and potassium thiocyanate were added, respectively, and the solutions were incubated for 24 h. After centrifugation to remove undissolved PAsc-PSFe, photos of the products were taken.

pH-Responsive magnetic resonance imaging (MRI) in solution

PAsc/Fe@Cy7QB was dispersed into acidic buffer solutions (pH = 7.4, 6.5, 5.5 and 5.0) separately to measure the T_1 relaxivity. Then, the shaken solution transferred into Sample Jet tubes for MRI scanning. In vitro MRI of PAsc/Fe@Cy7QB NPs at different concentrations was carried out on a Niumag 1.0 T whole-body magnetic resonance imaging scanner (NM42-040H-I, Niumag, China) at 35 °C. The array was embedded in a phantom consisting of a water tank to allow appropriate image acquisition. The following parameters were adopted: repetition time (TR) = 500 ms, echo time (TE) = 20 ms, number of averages = 3. The relaxivity value (r_1, L mM^{-1} s^{-1}) was calculated using T_1 measurements with a series of dilutions of the nanoparticle dispersions in 0.9% saline.

PA measurement in solution

For *in vitro* measurement, The PA signal intensity of PAsc/Fe@Cy7QB (50 μg/mL) incubated in solution with different H_2O_2 concentrations was recorded by a 10 MHz, 10 mJ cm^{-2}, 384-element ring ultrasound array. The multi-element transducer has a center frequency of 2.5 MHz with a nominal bandwidth of 70%.

Flow cytometry analysis

To study cell apoptosis induced by PAsc/Fe@Cy7QB, a flow cytometric assay involving Annexin V-FITC and PI costaining was carried out. The cells were harvested, rinsed in PBS, resuspended, and determined by flow cytometry (Becton Dickinson, Mountain View, CA, USA). All the experiments detected at least 10,000 cells, and the data were analyzed using the FCS Express V3. For live/dead assay, HepG2 cells were incubated with various samples for 4 h, following by staining with Calcein AM and PI by laser scanning confocal microscopy.
